# Pressure on bony prominences in the dorsal, lateral, and ventral decubitus: a clinical trial

**DOI:** 10.1590/1980-220X-REEUSP-2025-0073en

**Published:** 2025-08-29

**Authors:** Eline Lima Borges, Ana Luizi Perígolo Miranda, Perla Oliveira Soares de Souza, Mery Natali Silva Abreu, Josimare Aparecida Otoni Spira

**Affiliations:** 1 Universidade Federal de Minas Gerais, Escola de Enfermagem, Enfermagem Básica, Belo Horizonte, MG, Brazil.; 2 Universidade Federal de Minas Gerais, Escola de Enfermagem, Belo Horizonte, MG, Brazil.; 3 Universidade Federal de Minas Gerais, Escola de Enfermagem, Laboratório de Tecnologia e Inovação, Belo Horizonte, MG, Brazil.; 4 Universidade Federal de Minas Gerais, Escola de Enfermagem, Gestão em Saúde, Belo Horizonte, MG, Brazil.

**Keywords:** Pressure ulcer, Pressure, Supine Position, Nursing care, Clinical trial

## Abstract

**Objective:**

To identify pressure on bony prominences in the supine, lateral, and prone positions.

**Method:**

Phase I, experimental, non-randomized clinical study comparing interface pressures on bony prominences in three positions (dorsal, lateral, and ventral) in healthy individuals. Pressure was measured with the SR Soft Vision® sensor in all participants, positioned in the three decubitus positions and on 10 C-CORE® mattresses. Comparison of total pressure per decubitus position was performed by ANOVA.

**Results:**

448 assessments were performed in each decubitus position. The mean pressure was 652.3 (95% CI: 592.9–711.7) in the dorsal decubitus, 1.522.2 (95% CI: 1.423.0–1.621.4) in the lateral decubitus, and 313.2 (95% CI: 275.2–351.3) in the ventral decubitus. When stratified by sex (p < 0.001), in females, the mean pressure was 540.08 in the dorsal position, 1.200.9 in the lateral position, and 169.2 in the ventral position. In males, it was 761.3 in the dorsal position, 1.842.0 in the lateral position, and 455.3 in the ventral position. Stratification by weight also showed significant differences (p < 0.001) between decubitus positions.

**Conclusion:**

This study demonstrated significant variation in interface pressures between dorsal, lateral, and ventral decubitus positions. Registration in ReBEC: RBR-9vsvkn5.

## INTRODUCTION

Pressure injuries are one of the most frequent and costly adverse events in hospitals, but they are potentially preventable. They are internationally recognized as an important indicator of the quality of healthcare provision^(1)^. Their prevention is one of the patient safety goals and responsibilities of the multidisciplinary team at all levels of care in the health system^(2)^. When they develop, the cost of treatment can vary from protective coverage of bony prominences to a dynamic support surface and can reach several thousand euros, dollars, or reais^(3)^.

Pressure injuries are a major public health challenge, with variations between countries in terms of their global burden. Data from the 2019 Global Burden of Disease Study confirm that there has been an improvement in some indicators from 1990 to 2019. Globally in 2019, the number of prevalent cases of pressure injury was 0.85 million, with age-standardized prevalence, incidence, and disability-adjusted life years rates of 11.3, 41.8, and 1.7 per 100.000 population. The highest burden of pressure injuries was observed in elderly patients, especially in the 80−84 age group for men and 85−89 for women^(4)^. However, there were no statistically significant differences in prevalence, incidence, and years lived with disability between men and women. Significant regional differences were maintained between age-standardized years lived with disability and sociodemographic index^(4)^.

Seen from the patient’s perspective, pressure injuries cause physical, psychological, social, and economic losses. In terms of health services, they contribute to significant labor and financial losses. In addition, the cost of pressure injury prevention strategies remains statistically significant^(3)^. A systematic review study on the economic impact of pressure injuries in patients admitted to intensive care units identified that the four main sources of cost for prevention were: support surfaces, covers for bony prominences, human resources, especially nursing, and equipment for mobilization^(5)^.

The repositioning, at individualized times, of all individuals at risk of pressure injury is among the preventive measures^(6)^. This action aims to reduce the duration and magnitude of pressure exerted on vulnerable areas of the body and contribute to the comfort, hygiene, dignity, and functional capacity of the individual^(6)^.

The exposure of soft tissue to sustained deformations and stress concentrations, which normally occur in the vicinity of bony prominences, is linked to the position of the body (decubitus) on the support surface. The relationships between the levels of stress concentrations in soft tissue near bony prominences and the degree of involvement during weight-bearing postures are highly complex and may be linked to the occurrence of pressure injury. These interactions depend on the anatomical and geometric characteristics of the patient, the composition of the material and the structure of the support surface, as well as the specific position of the body^(7)^. Some studies have evaluated the intensity of pressure on bony prominences under the influence of body tilt^(8)^ and repositioning^(9,10)^.

There is no clear evidence on the best position and schedule for repositioning. A review found that, in general, the quality of studies is limited^(11)^. A meta-analysis did not identify conclusive evidence on the effectiveness of different frequencies and positions of decubitus change in preventing pressure injuries. However, changes every 3 to 4 hours may be more economical than every 2 hours. Regarding tilt positions (30° vs. 90°), the results were also inconclusive^(10)^. Although repositioning is necessary to prevent pressure injury, changing positioning less frequently may be a reality in clinical practice, given the properties of new mattress technologies and the lack of agreement in the evidence on repositioning^(11)^.

The usual patients’ position for distributing pressure evenly under patients is the flat dorsal decubitus (supine) position, which helps minimize shear and prevent pressure injury at the sacral/coccyx prominences^(12)^. However, patients should not remain in this position all the time. They are also placed in the lateral decubitus position and, in cases of severe respiratory failure, in the ventral decubitus (prone) position.

It is considered that most hospitals do not have sophisticated equipment for prevention, such as assisted rotation mattresses^(13)^ or wearable sensors to guide patient repositioning^(14)^. A popular strategy involves frequent rotation and repositioning of patients^(12)^. Therefore, knowledge about the value of pressure on bony prominences according to the decubitus position can contribute to the adoption of strategies for rotating and moving patients to prevent or reduce pressure injuries, especially for those with immobility or movement difficulties.

Given the lack of evidence on the intensity of pressure on bony prominences and its relationship with decubitus, the following research question arose: what is the pressure on bony prominences according to decubitus (dorsal, lateral, and ventral)? The answer to this question may support the revision of pressure injury prevention protocols in the management of repositioning patients at risk. Given the above, the study aims to identify the pressure on bony prominences in the dorsal, lateral, and ventral decubitus positions.

## METHOD

### Study Design

this is phase I of a clinical study, of the experimental factorial type, non-randomized, comparative, without masking to elucidate the hypothesis: the ventral, lateral, and dorsal decubitus positions have the same pressure on the bony prominences (interface pressure). For the communication of the study, the assumptions of the *Consolidated Standards of Reporting Trials* (CONSORT) statement^(15)^ were considered. The study is registered in the Brazilian Clinical Trials Registry (ReBEC), with primary identifier RBR-9vsvkn5.

### Participants

The eligibility criteria were: participants of both sexes, aged over 18 years, with all limbs intact, independent movement, allowing the person to move autonomously and without restrictions, and weighing up to 180 kg. The exclusion criteria were: report of urinary or fecal incontinence, presence of marked spinal deviation on inspection, skin diseases of the desquamative type, skin wounds, or scars in the gluteal or trochanteric region.

The study was conducted at the Technology and Innovation Laboratory (LABTEC C-CORE/UFMG), located at the School of Nursing of the Federal University of Minas Gerais. At LABTEC, a setting similar to a hospital ward was created with a Qualitas Plus™ Paramount bed (A5 series) equipped with a C-CORE^®^ mattress, whose raw material is produced by C-CORE Brasil. The mattress is composed of a flexible, three-dimensional reticulated blanket made of high-density long-chain polyethylene filaments (3D LOOP), whose aerated characteristic can enhance skin microclimate control (temperature and humidity). The organization of the filaments creates a structure that allows for a high level of ventilation. The environment during data collection was maintained at a temperature of 23°C.

Recruitment took place from January to June 2024, through an invitation in which interested parties were asked to fill out a form on Google Forms with personal information and answer questions according to the eligibility criteria. In addition, they scheduled the day and time for data collection.

### Outcomes

The study required three interventions: dorsal, ventral, and lateral decubitus, forming three distinct groups for measuring the outcome, interface pressure on bony prominences.

Data collection took place from June to December 2024. Participants received explanations about the study and had the opportunity to clarify any doubts. In addition, body weight was measured.

The dependent variable (outcome) was interface pressure and the independent variable was the type of decubitus (dorsal, lateral, and ventral). The following variables were also considered: age, sex, and weight (in kilograms) categorized as < 50 kg, ≥ 50 kg and ≤ 80 kg, > 80 kg and ≤ 100 kg, > 100 kg and ≤ 180 kg.

To assess interface pressure, an SR Soft Vision^®^ sensor was used, which is a blanket made of tiny cells that capture the magnitude of pressure and the area of contact with the person›s body surface, converting it into data using Windows^®^-compatible software. The blanket was placed on each mattress and the sensor cable, connected to the computer, captured the image. For this study, pressure points equal to and above 79 mmHg were considered high. To calculate the total pressure, all high pressure points were added together.

Ten types of 15 cm high C-CORE^®^ mattresses with different densities were used for the study. The results of the pilot test in the dorsal, lateral, and ventral positions, involving all 10 mattresses, allowed the mattresses to be considered as a single variable, without categories.

Each participant wore light clothing, such as leggings and a T-shirt, without pockets, ties, or seams to avoid pressure points. The person was placed in a lying position without a pillow, and the pressure was assessed, especially in areas of bony prominences. All participants were positioned in the three decubitus positions and on the 10 C-CORE^®^ mattresses, following the schedule at LABTEC.

In the dorsal (supine) decubitus, pressure was measured in the head, back, posterior cubital, coccygeal, and heel regions. In the lateral decubitusn, the regions of the face, back, trochanteric, patella, and lateral foot were measured. In the ventral (prone) decubitus, the regions of the ear, face, posterior cubital, breast, pelvis, patella, and toes were evaluated. The positions were parameterized with the aid of a goniometer and tape measure to maintain the angulation of certain joints and body alignment. Body alignment enabled each individual to reproduce the position guided by the evaluator, so that there was no deviation from the positioning. Data collection with each participant in the three decubitus positions (dorsal, lateral, and ventral) took around 30 minutes. The photo generated by the software sensor was recorded one minute after each positioning.

### Sample Size

In this study, the unit of analysis was the decubitus position. For the sample calculation, the parameters estimated in the previously conducted pilot study were considered, with a difference in pressure between the positions (dorsal, lateral, ventral) of 60% (effect size of 0.60). For a significance level of 5% and power of 80%, a sample of 45 individuals per group was estimated, with each individual being tested on 10 different mattresses, resulting in a total of 450 evaluations. The calculation was performed using the GPower 3.1.9.7 program.

The sample size, calculated from the pilot results, was higher than the reference for phase I of the clinical study, in which the developed product is tested on a group of 20 to 100 volunteers without comorbidities or complications, with the aim of evaluating the safety, compatibility, and efficacy of the product^(16)^.

### Statistical Methods

The study used SPSS version 21.0 software for data analysis. A comparison of total pressure by decubitus was performed, considering the C-CORE^®^ mattress, using a one-way analysis of variance (ANOVA). In this case, the outcome considered was total pressure and the analysis variable (study group) was the type of decubitus (dorsal, lateral, and ventral). This analysis was stratified by gender and body weight. Tukey›s multiple comparison test was performed when significant differences were detected between the groups. In all analyses, a significance level of 5% was considered.

### Ethical Aspects

The study complied with Resolution No. 466/12 of the National Health Council. It was approved by the Research Ethics Committee under opinion No. 6.853.674, Certificate of Presentation for Ethical Review (CAAE) 78828624.3.0000.5149. It should be noted that participation was conditional upon the participant signing an Informed Consent Form.

In accordance with Open Science principles, we hereby inform that the data are not currently available, as the study is the result of a Research, Development, and Innovation Project (PD&I), a public-private partnership with the company C-CORE Brasil.

## RESULTS

**Figure 1:**
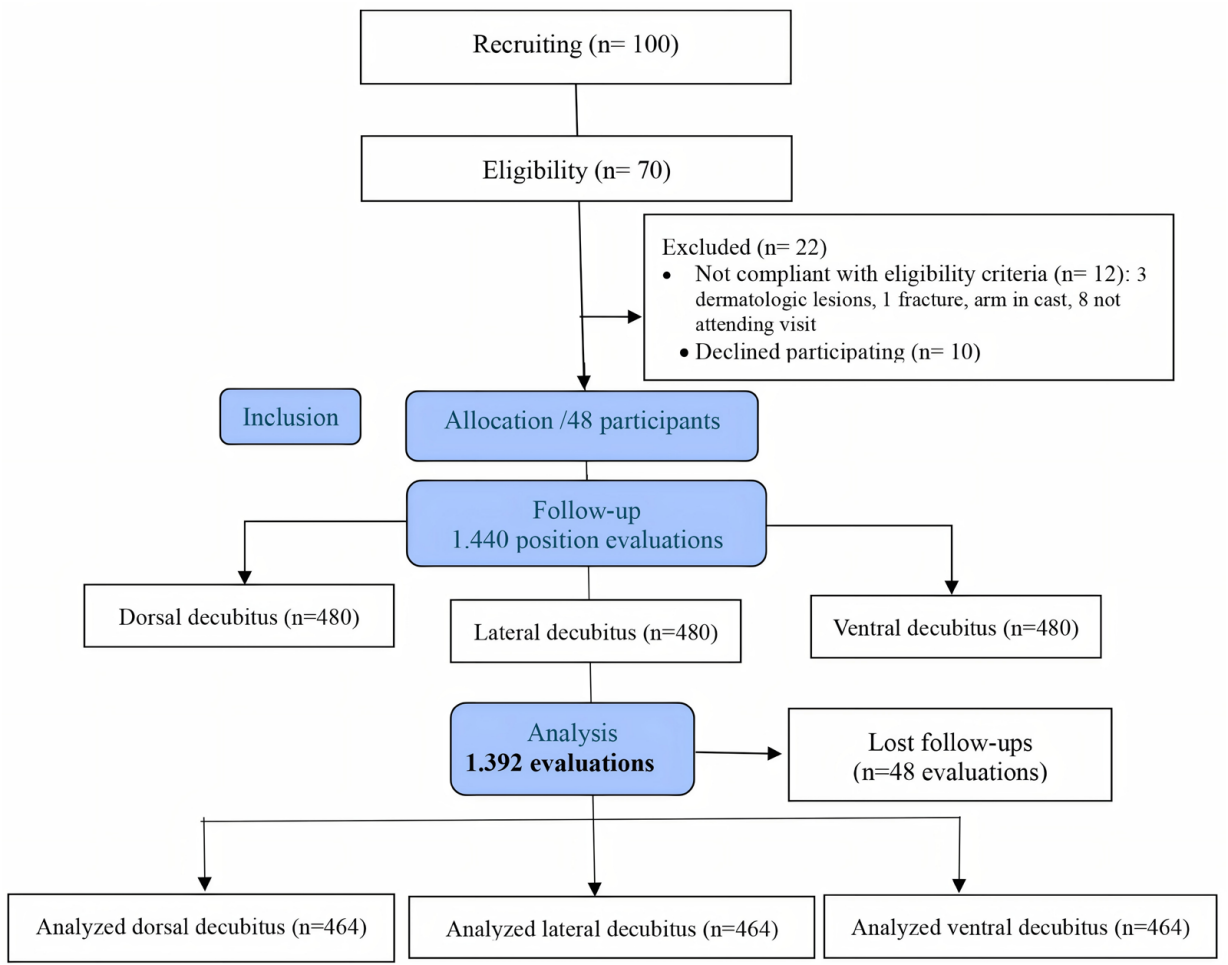
*Consort* diagram. Belo Horizonte, MG, Brazil, 2024.

Figure 1 shows the Consort diagram for the selection of study participants^(15)^. One hundred participants were recruited for the study, with 48 allocated. The study resulted in 464 assessments in each decubitus position, considering that this is the unit of analysis, totaling 1.392 decubitus positions.

Regarding the characteristics of the participants, 24 (50%) were male and 24 (50%) were female. The average weight was 69.7 kg (SD 24.3), ranging from 40.0 kg to 113.5 kg. The minimum age was 19 years and the maximum age was 53 years, with an average age of 32.2 years (SD 11.7).

**Figure 2:**
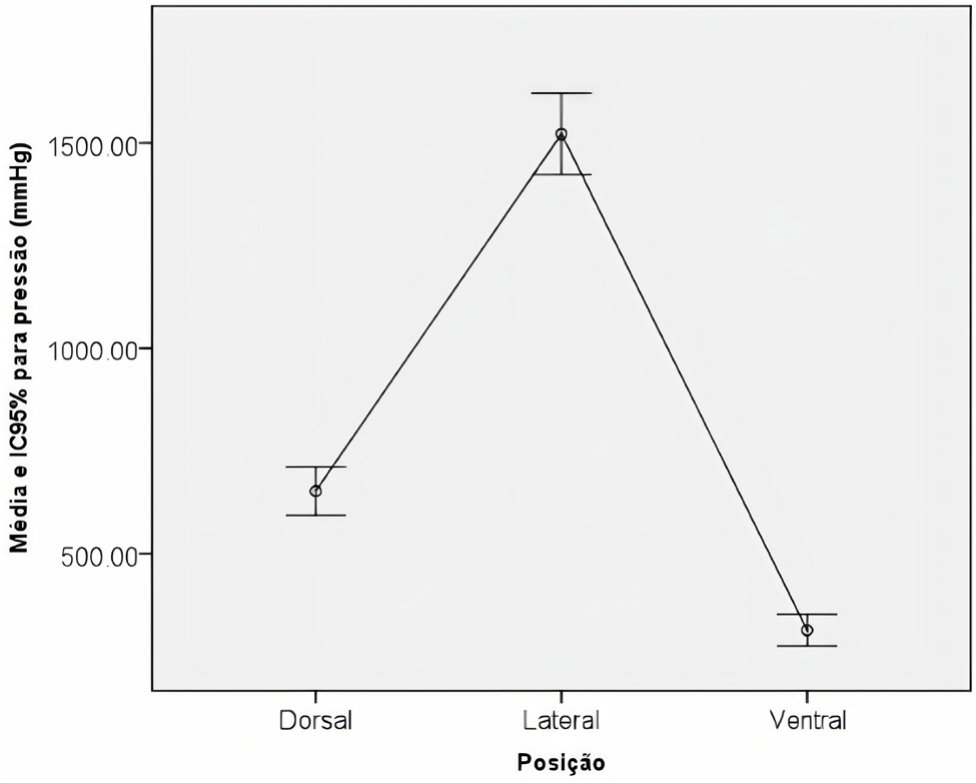
Graph of average pressure values by position.

When comparing the average pressure exerted according to the dorsal, lateral, and ventral decubitus positions (Figure 2), the average pressure in the dorsal position was 652.3 ± 30.2 (95% confidence interval [CI]: 592.9−711.70), lateral of 1.522.2 ± 50.5 (95% CI: 1.423.0−1.621.4) and ventral of 313.2 ± 19.4 (95% CI: 275.2−351.3).

Regarding multiple comparisons of total pressure between the analyzed decubitus positions, considering multiple comparison tests (Table 1), there was a significant difference between them (p-value <0.001).

**Table 1:** Multiple comparisons of pressure between the analyzed decubitus positions – Belo Horizonte, MG, Brazil, 2024.

Decubitus		Effect size	Standard error	P-value*	CI 95%^#^	
					Lower limit	Upper limit
Dorsal	Lateral	−869.9	50.5	0.000	−988.4	−751.4
	Ventral	339.0	50.4	0.000	220.7	457.4
Lateral	Dorsal	869.9	50.5	0.000	751.4	988.4
	Ventral	1.208.9	50.5	0.000	1.090.5	1.327.5
Ventral	Dorsal	−339.0	50.4	0.000	−457.4	−220.7
	Lateral	−1.209.0	50.5	0.000	−1.327.5	−1.090.5

**Caption:** *Tukey test.

^#^95% confidence interval for effect size.

**Figure 3:**
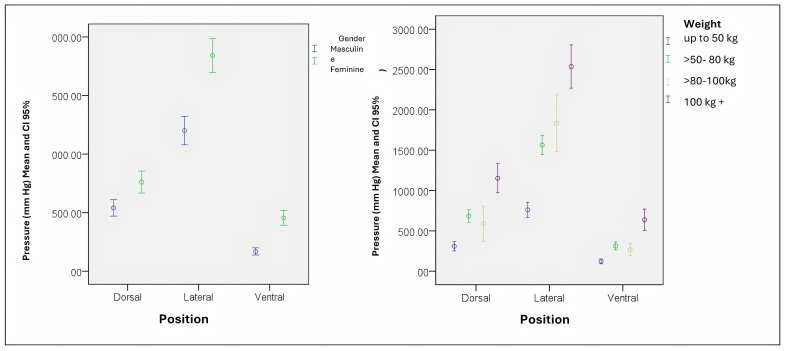
Average pressure values by decubitus position stratified by gender and weight.

When stratified by sex (Figure 3), the difference between decubitus positions remained significant (p-values <0.001). In females, the mean pressure in the dorsal position was 540.08 ± 35.7 (95% CI: 470.4−611.2). In the lateral decubitus position, the mean pressure was 1.200.9 ± 61.7 (95% CI: 1.079.2−1.322.6), while in the ventral position it was 169.2 ± 15.5 (95% CI: 138.7−199.6). For the male group, the mean pressure in the dorsal position was 761.3 ± 47.5 (95% CI: 667.8−854.9). In the lateral decubitus position, the mean was 1.842.0 ± 73.8 (95% CI: 169.5−1.987.6) and in the ventral position it was 455.3 ± 32.7 (95% CI: 390.9−519.7).

The magnitude of the differences between the lateral position and the other positions maintains the statistical difference in relation to sex, and the greater the weight, the greater the pressures in the lateral decubitus position in relation to the others.

In multiple comparison tests (Table 2), there was a significant difference between all decubitus positions for both genders. However, it can be observed that the magnitude of the differences between dorsal and lateral decubitus, and ventral and lateral decubitus are greater among males (effect of 1.080.7 and 1.386.7, respectively) than among females (effect of 660.1 and 1.031.7, respectively).The difference between decubitus positions remains significant (p-values <0.001) in the weight stratification, with lower pressure levels for the ventral decubitus position and higher for the lateral decubitus position for the four weight categories (Table 2).

**Table 2:** Multiple comparisons of pressure (mmHg) between decubitus positions by sex and weight – Belo Horizonte, MG, Brazil, 2024.

Variables	Position		Effect Size (mmHg)	Standard error	P-value*	CI 95%^#^	
						Lower limit (mmHg)	Upper limit (mmHg)
**Gender**							
Feminine	Dorsal	Lateral	−660.1	59.6	0.000	−800.2	−520.0
		Ventral	371.6	59.6	0.000	231.5	511.7
	Lateral	Dorsal	660.1	59.6	0.000	520.0	800.2
		Ventral	1031.7	59.6	0.000	891.8	1.171.6
	Ventral	Dorsal	−371.6	59.6	0.000	−511.7	−231.5
		Lateral	−1031.7	59.6	0.000	−1171.6	−891.8
Masculine	Dorsal	Lateral	−1080.7	76.3	0.000	−1259.9	−901.5
		Ventral	306.0	76.1	0.000	127.2	484.8
	Lateral	Dorsal	1080.7	76.3	0.000	901.5	1.259.9
		Ventral	1386.7	76.4	0.000	1207.3	1.566.1
	Ventral	Dorsal	−306.0	76.1	0.000	−484.8	−127.2
		Lateral	−1386.7	76.4	0.000	−1.566.1	−1.207.3
**Weight**							
< 50 Kg	Dorsal	Lateral	−451.6	47.3	0.000	−563.0	−340.3
		Ventral	187.4	47.1	0.000	76.7	298.1
	Lateral	Dorsal	451.6	47.3	0.000	340.3	563.0
		Ventral	639.1	47.2	0.000	528.0	750.2
	Ventral	Dorsal	−187.4	47.1	0.000	−298.1	−76.7
		Lateral	−639.1	47.2	0.000	−750.2	−528.0
≥ 50Kg to ≤80Kg	Dorsal	Lateral	−879.9	62.4	0.000	−1.026.5	−733.3
		Ventral	370.4	62.5	0.000	223.5	517.3
	Lateral	Dorsal	879.9	62.4	0.000	733.3	1.026.5
		Ventral	1250.3	62.5	0.000	1.103.5	1.397.0
	Ventral	Dorsal	−370.4	62.5	0.000	−517.3	−223.5
		Lateral	−1250.3	62.5	0.000	−1.397.0	−1.103.5
> 80 Kg to ≤ 100 Kg	Dorsal	Lateral	−1244.1	165.9	0.000	−1.640.2	−847.9
		Ventral	323.1	164.4	0.128	−69.5	715.6
	Lateral	Dorsal	1244.1	165.9	0.000	847.9	1.640.2
		Ventral	1567.1	165.9	0.000	1.171.0	1.963.3
	Ventral	Dorsal	−323.1	164.4	0.128	−715.6	69.5
		Lateral	−1567.1	165.9	0.000	−1963.3	−1171.0
> 100 Kg to ≤ 180kg	Dorsal	Lateral	−1384.3	143.5	0.000	−1722.6	−1046.0
		Ventral	514.8	143.5	0.001	176.5	853.1
	Lateral	Dorsal	1384.3	143.5	0.000	1046.0	1722.6
		Ventral	1899.1	143.5	0.000	1560.7	2237.4
	Ventral	Dorsal	−514.8	143.5	0.001	−853.1	−176.5
		Lateral	−1899.1	143.5	0.000	−2237.4	−1560.7

**Caption:** *Tukey test.

^#^95% confidence interval for mean.

## DISCUSSION

There was a significant difference in pressure between the dorsal, lateral, and ventral positions, with lower pressure levels in the ventral position. The lateral position generated the highest pressures on bony prominences, especially in the trochanteric region. Understanding the pressure resulting from positioning can support strategies to minimize tissue deformities, reducing peak tension and stress values or decreasing exposure time and, consequently, preventing pressure injuries.

The results of a systematic review indicate that more frequent repositioning and the use of a rotation team reduce the incidence of pressure injuries. However, there was low certainty of the evidence, so the results should be interpreted with caution^(17)^. These results, combined with the finding that the lateral position causes greater pressure on the bony prominences of healthy people, can assist clinical practitioners in determining the length of time a patient should remain in this position. Decision-making should consider the ability of the support surface (mattress) to redistribute pressure.

Under normal conditions, maximum capillary pressure is approximately 20 mmHg and mean tissue pressure varies between 16 and 33 mmHg. However, when higher pressures are triggered by long periods of the body in the same position, there is an increased risk of developing tissue necrosis. Prolonged compression of tissues leads to reduced blood flow, causing microcirculatory damage and decreased oxygen delivery, resulting in ischemia and pressure injury^(9)^. When comparing the average pressure exerted according to the decubitus positions, the average pressure in the ventral position was 313.2 mmHg, in the dorsal position 652.3 mmHg, and in the lateral position 1.522.2 mmHg. These values are higher than the maximum capillary pressure. Therefore, it is necessary to ensure that the decubitus position and support surface are changed as needed.

High tissue deformations result in cellular damage at a microscopic level in just a few minutes, although it may take hours of sustained loading for the damage to become clinically visible. Superficial damage to the skin appears to be caused mainly by excessive tension exposure. However, deeper pressure injuries result predominantly from high pressures in combination with shearing over bony prominences^(18)^.

Over the past two decades, it has become evident that sustained deformation of cells leads directly to cell damage and death. The proposed pathways include changes in the cytoskeleton and an increase in cell membrane permeability, exceeding the threshold for normal cell homeostasis. Under high tissue deformation, when the individual damage threshold is exceeded, this process is believed to be rapid, occurring within minutes^(19)^.

Considering all compromised tissue layers, evidence indicates that signs of pressure injury development may be detectable in all involved tissues, including skin, subcutaneous fat, or muscle^(20)^. The anatomical location, morphology (e.g., bone geometry, tissue thickness), the properties of soft and rigid tissues, the degree of tissue deformation, the skin microclimate, individual tolerance, and repair capacity determine whether damage thresholds are exceeded, leading to deeper or more superficial pressure injuries^(21)^.^.^

In 2005, researchers investigated four preventive regimens involving frequent (2, 3 hours) or less frequent (4, 6 hours) changes in position on different mattresses. Over 28 days, four different schemes were used: changing every 2 hours on a standard institutional mattress, changing every 3 hours on a standard mattress, changing every 4 hours on a viscoelastic foam mattress, and changing every 6 hours on a viscoelastic foam mattress. These groups had 65 or 67 patients. The researchers concluded that changing position on the memory foam mattress every 4 hours resulted in a significant reduction in the number of pressure injuries^(22)^. However, a recent Cochrane systematic review showed that repositioning may be useful in reducing the risk of pressure injuries. However, its effectiveness depends on factors such as the support surface used and the patient’s clinical condition. In addition, it was not possible to determine with certainty which time interval is most effective for repositioning, whether every 2, 3, or 4 hours. Furthermore, when it comes to lateral decubitus, there is no strong evidence that a 30° or 90° tilt is an effective strategy for reducing pressure in vulnerable areas^(10)^.

In the present study, the magnitude of the differences between the supine and lateral decubitus positions and between the ventral and lateral decubitus positions is greater among men than among women, which may be justified by the anatomical difference in the bone structure of each sex. This fact is related to the result of a study that identified that the ideal inclination for repositioning may depend on sex and body mass index^(8)^.

In contrast, studies show that patients positioned in the lateral decubitus position at a 90° angle to the support surface for more than 2 hours can reduce blood flow to levels close to anoxia, while a 30° inclination improves oxygenation and reduces complications^(9)^. Although patient repositioning is applied in everyday hospital and out-of-hospital care and recommended by good practice guidelines, there is still a need for robust studies on the best time interval for patient repositioning.

Anatomical differences between men and women show that men may be more prone to pressure injuries due to the greater concentration of forces in specific areas of the pelvis. The male pelvis has a more closed subpubic angle and closer acetabula, resulting in a more compact and rigid structure. This configuration can concentrate body weight in smaller areas, such as the ischial tuberosities, generating greater pressure in these regions, especially during sitting or lateral decubitus^(23)^. Although sex may influence anatomical and physiological characteristics, it should be considered that it is not an isolated determining factor for the onset of pressure injuries^(24)^. The discrepancies reinforce the need for further studies on the subject.

A study conducted with healthy individuals found that those with obesity exerted greater pressure on the trochanteric region at a body inclination of 30°, when compared to individuals who were overweight, normal weight, and underweight, respectively^(8)^. The study identified that body weight is significantly related to interface pressure in the lateral decubitus position. The greater the weight, the greater the pressure on the bony prominences. The finding supports nurses in determining the time for repositioning. Therefore, the time to keep the patient in the lateral position should be shorter for those with higher body weight, since the possibility of pressure injury increases proportionally with weight. Given these findings, it is suggested that, in addition to gender, the influence of body weight on the pressure exerted should be considered, as this may impact the formulation of strategies for the prevention of pressure injuries.

In the study, the ventral position was the one with the least pressure on the bony prominences. The ventral decubitus, known as the prone position, was evident in the management of acute respiratory distress syndrome in patients with COVID-19 during the pandemic. Its main benefit was a reduction in mortality. However, this position also had some disadvantages. One of these was an increased risk of pressure injury in atypical body regions due to continuous contact with the support surface, such as the face (forehead, nose, cheek, lip, chin), chest, knee, pretibial region, and toes^(25)^.

In addition to repositioning, the support surface should be adjusted to meet the individual needs of the patient, considering factors such as skin condition and risk stratification for injuries. However, there is a lack of evidence to support a support surface as the gold standard for correct weight distribution and pressure relief^(26)^. Identifying the optimal frequency of decubitus change and repositioning is critical for the prevention of pressure injuries in healthcare settings. In addition, nursing researchers should be encouraged to conduct further research to investigate the factors that influence the effectiveness of change frequency in the development of pressure injuries^(12)^.

This is a non-randomized factorial experimental design study, which may introduce biases, as the allocation of participants to different positions was not random, which may affect internal validity. As the study was conducted in a laboratory setting with 10 mattresses of the same technology, the findings may not be directly applicable to other populations, clinical contexts, or different support surfaces. It should be noted that the interface pressure was assessed exclusively with SR Soft Vision® sensor software, which may limit comparison with other technologies or methods of measuring the patient-mattress interface. The study analyzed 1.392 positions obtained from 48 participants, which may compromise the representativeness and correlation between repeated measurements within the same individual. Although the study stratified by age, gender, and body weight, other variables, such as muscle tone, body mass index, or body fat distribution, were not considered, which may influence the results.

The intervention (type of decubitus) presented relevant results for professionals, especially nurses, in defining the decubitus for positioning patients at risk of pressure injury, especially those with impaired mobility. The findings support nursing prescriptions regarding decubitus, and greater attention should be given to lateral decubitus, as it triggers greater pressure at the interface of bony prominences.

The results support the need to review the time for repositioning men and those with higher body weight, regardless of the support surface in use. Finally, it is recommended that the nursing team use risk assessment scales for pressure injuries, establish assertive interventions, and monitor the occurrence of pressure injuries, such as instruments that can assess the quality and effectiveness of approaches in the routine prevention of pressure injuries. Another potential of the study is the use of the results to review pressure injury prevention protocols, with emphasis on reviewing the repositioning time considering the dorsal, lateral, and ventral decubitus positions, according to the patients’ risk score, gender, and body weight.

The study provides support for future clinical investigations in a hospital setting, with the aim of evaluating, in a real-world context, the pressures exerted on the lateral, ventral, and dorsal decubitus positions of patients at risk for developing pressure injuries.

## CONCLUSION

The study refuted the hypothesis that the ventral, lateral, and dorsal decubitus positions exert the same pressure on bony prominences (interface pressure), since there were lower pressure levels for the ventral decubitus position and higher levels for the lateral decubitus position in both sexes and body weights. This fact supports the choice of the ventral decubitus position for patients with restricted repositioning. The magnitude of the differences between the supine and lateral decubitus positions is greater among men, and the magnitude of the differences between the decubitus positions increases with weight, i.e., the greater the weight, the greater the pressure in the lateral position compared to the others. The findings reinforce the need to review decubitus change protocols, minimizing pressure peaks in vulnerable areas.

## Data Availability

The data supporting this study are confidential and not publicly available, as they originate from a Research, Development, and Innovation (RD&I) Project conducted through a public- private partnership with the company C-CORE Brasil.
