# Supplementary material for: Pressure on bony prominences in the dorsal, lateral, and ventral decubitus: a clinical trial
Source: Rev Esc Enferm USP. 2025 Aug 29;59:e20250073. doi: 10.1590/1980-220X-REEUSP-2025-0073en (PMC12395604; doi:10.1590/1980-220X-REEUSP-2025-0073en)
Supplement: Translation

# Translation: Pressão nas proeminências ósseas conforme os decúbitos dorsal, lateral e ventral: ensaio clínico

**DOI:** 10.1590/1980-220X-REEUSP-2025-0073pt

**Authors:** Eline Lima Borges, Ana Luizi Perígolo Miranda, Perla Oliveira Soares de Souza, Mery Natali Silva Abreu, Josimare Aparecida Otoni Spira

**Affiliations:** 1 Universidade Federal de Minas Gerais, Escola de Enfermagem, Enfermagem Básica, Belo Horizonte, MG, Brasil.; 2 Universidade Federal de Minas Gerais, Escola de Enfermagem, Belo Horizonte, MG, Brasil.; 3 Universidade Federal de Minas Gerais, Escola de Enfermagem, Laboratório de Tecnologia e Inovação, Belo Horizonte, MG, Brasil.; 4 Universidade Federal de Minas Gerais, Escola de Enfermagem, Gestão em Saúde, Belo Horizonte, MG, Brasil.

**Keywords:** Úlcera por Pressão, Pressão, Decúbito Dorsal, Cuidados de Enfermagem, Ensaio Clínico

## Abstract

**Objetivo:**

Identificar a pressão nas proeminências ósseas nos decúbitos dorsal, lateral e ventral.

**Método:**

Estudo clínico de fase I, experimental e não randomizado, que comparou as pressões de interface nas proeminências ósseas em três decúbitos (dorsal, lateral e ventral) em indivíduos sadios. A pressão foi medida com o sensor SR Soft Vision® em todos os participantes, posicionados nos três decúbitos e sobre 10 colchões C-CORE^®^. A comparação da pressão total por decúbito foi realizada por ANOVA.

**Resultados:**

Foram realizadas 448 avaliações em cada decúbito. A pressão média foi 652,3 (IC 95%: 592,9−711,7) no decúbito dorsal, 1.522,2 (IC 95%: 1.423,0−1.621,4) no lateral e 313,2 (IC 95%: 275,2−351,3) no ventral. Ao estratificar por sexo (p < 0,001), no feminino, a média de pressão foi 540,08 dorsal, 1.200,9 lateral e 169,2 ventral. No sexo masculino, foi 761,3 dorsal, 1.842,0 lateral e 455,3 ventral. A estratificação por peso também apresentou diferenças significativas (p < 0,001) entre os decúbitos.

**Conclusão:**

Este estudo demonstrou variação significativa nas pressões de interface entre os decúbitos dorsal, lateral e ventral. Registro no ReBEC: RBR-9vsvkn5.

## INTRODUÇÃO

As lesões por pressão são um dos eventos adversos mais frequentes e dispendiosos em hospitais, mas são potencialmente evitáveis. São reconhecidas internacionalmente como um indicador importante da qualidade da prestação de cuidados de saúde^(1)^. A sua prevenção é uma das metas de segurança do paciente e responsabilidade da equipe multidisciplinar em todos os níveis de atenção do sistema de saúde^(2)^. Quando elas se desenvolvem, o custo do tratamento pode variar de uma cobertura de proteção das proeminências ósseas para uma superfície de suporte dinâmica e pode atingir vários milhares de euros, dólares ou reais^(3)^.

As lesões por pressão são um grande desafio de saúde pública com variações entre os países em relação à sua carga em nível global. Os dados do *Global Burden of Disease Study* 2019 confirmam que houve melhora de alguns indicadores de 1990 a 2019. Globalmente em 2019, o número de casos prevalentes de lesão por pressão foi de 0,85 milhões, a prevalência padronizada por idade, incidência e taxas de anos vividos com incapacidade foram 11,3, 41,8 e 1,7 por 100.000 habitantes. A maior carga de lesão por pressão foi observada em pacientes idosos, especialmente na faixa etária de 80 a 84 anos para homens e 85 a 89 para mulheres^(4)^. Contudo, não houve diferenças estatisticamente significativas para prevalência, incidência e anos vividos com incapacidade entre homens e mulheres. As diferenças regionais significativas foram mantidas entre anos vividos com incapacidade, padronizados por idade e índice sociodemográfico^(4)^.

Da perspectiva do paciente, as lesões por pressão causam perdas físicas, psicológicas, sociais e econômicas. Em termos de serviços de saúde, elas contribuem para perdas trabalhistas e financeiras significativas. Além disso, o custo das estratégias de prevenção de lesão por pressão mantém diferenças estatisticamente significativas^(3)^. Estudo de revisão sistemática sobre o impacto econômico das lesões por pressão em pacientes internados em unidades de terapia intensiva identificou que as quatro principais fontes de custo para a prevenção foram: superfícies de suporte, coberturas para proeminências ósseas, recursos humanos, especialmente de enfermagem e equipamento para a mobilização^(5)^.

O reposicionamento, em horário individualizado, de todos os indivíduos com risco de ocorrência de lesão por pressão está dentre as medidas de prevenção^(6)^. Esta ação visa reduzir a duração e a magnitude da pressão exercida sobre áreas vulneráveis do corpo e contribuir para o conforto, a higiene, a dignidade e a capacidade funcional do indivíduo^(6)^.

A exposição do tecido mole às deformações sustentadas e concentrações de estresse, que normalmente ocorrem nas proximidades das proeminências ósseas tem vinculação com a posição do corpo (decúbito) sobre a superfície de suporte. As relações entre os níveis de concentrações de estresse do tecido mole perto de proeminências ósseas e o grau de envolvimento durante posturas de sustentação de peso são altamente complexas e podem ter ligação com a ocorrência de lesão por pressão. Essas interações dependem das características anatômicas e geométricas do paciente, da composição do material e da estrutura da superfície de suporte, além da posição específica do corpo^(7)^. Alguns estudos avaliaram intensidade da pressão nas proeminências ósseas sob a influência da inclinação do corpo^(8)^ e o seu reposicionamento^(9,10)^.

Não há evidências claras sobre qual posição e cronograma de reposicionamento são os melhores. Uma revisão constatou que, no geral, a qualidade dos estudos é limitada^(11)^. Uma metanálise não identificou evidência conclusiva sobre a eficácia de diferentes frequências e posições de mudança de decúbito na prevenção de lesões por pressão. Entretanto, mudanças a cada 3h ou 4h podem ser mais econômicas do que a cada 2h. Em relação às posições de inclinação (30° vs. 90°), os resultados também foram inconclusivos^(10)^. Embora o reposicionamento seja necessário para prevenir lesão por pressão, mudar o posicionamento com menos frequência pode ser uma realidade na prática clínica, dadas as propriedades das novas tecnologias dos colchões e a falta de concordância nas evidências sobre o reposicionamento^(11)^.

A posição habitual dos pacientes para distribuir a pressão é a posição dorsal plana, que ajuda a minimizar o cisalhamento e prevenir lesão por pressão nas proeminências sacro/cóccix^(12)^. Contudo, os pacientes não devem ficar todo tempo nesta posição. Eles também são colocados na posição lateral e nos casos de insuficiência respiratória grave, na posição prona.

Considera-se que não é a realidade de grande parte das instituições hospitalares contar com equipamentos sofisticados para a prevenção, como por exemplo, colchões de rotação assistida^(13)^ ou o uso de sensores vestíveis para orientar o reposicionamento de pacientes^(14)^. Uma estratégia popular envolve a rotação e o reposicionamento frequentes dos pacientes^(12)^. Por isso, o conhecimento sobre o valor da pressão nas proeminências ósseas conforme o decúbito pode contribuir para adoção de estratégias de rotação e movimentação dos pacientes na prevenção ou redução de lesões por pressão, especialmente para aqueles com imobilidade ou dificuldades de movimento.

Diante da insuficiência de evidência sobre a intensidade da pressão nas proeminências ósseas e a sua relação com o decúbito, suscitou a questão de pesquisa: qual é a pressão nas proeminências ósseas conforme o decúbito (dorsal, lateral e ventral)? A resposta à questão do estudo pode amparar a revisão de protocolos de prevenção de lesão por pressão no manejo do reposicionamento de paciente em risco. Diante do exposto, o estudo tem o objetivo de identificar a pressão nas proeminências ósseas nos decúbitos dorsal, lateral e ventral.

## MÉTODO

### Desenho do Estudo

Trata-se da fase I de estudo clínico, do tipo experimental fatorial, não randomizado, comparativo, sem mascaramento para elucidar a hipótese: os decúbitos ventral, lateral e dorsal têm a mesma pressão nas proeminências ósseas (pressão de interface). Para a comunicação do estudo foram considerados os pressupostos do enunciado *Consolidated Standards of Reporting Trials* (CONSORT)^(15)^. O estudo apresenta Registro Brasileiro de Ensaios Clínicos (ReBEC), com identificador primário RBR-9vsvkn5.

### Participantes

Os critérios de elegibilidade foram: participantes de ambos os sexos, idade superior a 18 anos, presença de todos os membros do corpo, independência de movimento, permitindo que a pessoa se mova de forma autônoma e sem restrições e peso de até 180 kg. Os critérios de exclusão foram: relato de incontinência urinária ou fecal, presença de desvio acentuado da coluna à inspeção, doenças dermatológicas do tipo descamativa, feridas cutâneas ou cicatrizes na região glútea ou trocantérica.

O estudo foi realizado no Laboratório de Tecnologia e Inovação (LABTEC C-CORE/UFMG), localizado na Escola de Enfermagem da Universidade Federal de Minas Gerais. No LABTEC foi criado um cenário semelhante à unidade de internação com cama hospitalar da marca Qualitas Plus™ Paramount bed - série A5, com colchão C-CORE^®^, cuja matéria prima é produzida pela empresa C-CORE Brasil. O colchão é composto por manta reticulada tridimensional de filamentos de polietileno de alta densidade de cadeia longa (3D LOOP), flexível, cuja característica aerada pode potencializar o controle do microclima da pele (temperatura e umidade). A organização dos filamentos cria estrutura que permite alto nível de ventilação. O ambiente durante a coleta de dados foi mantido na temperatura de 23°C.

O recrutamento ocorreu de janeiro a junho de 2024, por meio de um convite, no qual o interessado deveria preencher um formulário no *Google Forms* com informações pessoais e responder a perguntas conforme os critérios de elegibilidade. Ademais, fez o agendamento do dia e horário para a coleta de dados.

### Desfecho

O estudo demandou três intervenções: decúbito dorsal, decúbito ventral e decúbito lateral, formando três grupos distintos para a mensuração do desfecho, pressão de interface nas proeminências ósseas.

A coleta de dados foi no período de junho a dezembro de 2024. Os participantes receberam explicações sobre o estudo e tiveram a oportunidade de esclarecer eventuais dúvidas. Além disso, foi mensurado o peso corporal.

A variável dependente (desfecho) foi pressão de interface e a variável independente o tipo de decúbito (dorsal, lateral e ventral). Também foram consideradas as variáveis: idade, sexo e peso (em quilos) categorizado em < 50 Kg, ≥ 50 Kg e ≤ 80 Kg, > 80 Kg e ≤ 100 Kg, > 100 Kg e ≤ 180kg.

Para avaliação da pressão de interface foi utilizado um sensor SR Soft Vision^®^, que é uma manta feita de minúsculas células que captam a magnitude da pressão e a área de contato da superfície do corpo da pessoa, convertendo em dados por um software compatível com Windows^®^. A manta foi colocada sobre cada colchão e o cabo do sensor, conectado no computador, fazia a captação da imagem. Para este estudo foram considerados os pontos de pressão igual e acima de 79 mmHg, classificados como elevados. Para o cálculo da pressão total foram somados todos os pontos de pressão elevada.

Para o estudo foram utilizados 10 tipos de colchões C-CORE^®^ de altura de 15cm, com densidade distinta. O resultado do teste piloto nos decúbitos dorsal, lateral e ventral, envolvendo os 10 colchões, permitiu considerar os colchões como variável única, sem categorias.

Cada participante vestiu roupa leve, tipo *legging* com camiseta, sem bolso ou laço ou costura para evitar pontos de pressão. A pessoa foi colocada na posição deitada sem o travesseiro e a pressão foi avaliada, especialmente nas áreas das proeminências ósseas. Todos os participantes foram posicionados nos três decúbitos e sobre os 10 colchões da tecnologia C-CORE^®^, respeitando a sequência de agendamento no LABTEC.

Na posição dorsal mediu-se a pressão nas regiões da cabeça, do dorso, cubital posterior, coccígea e calcanhar. Na posição lateral foram as regiões da face, do dorso, trocantérica, patela e lateral do pé. Na posição ventral (prona) avaliou-se as regiões da orelha, da face, cubital posterior, mama, pelve, patela e dedos do pé. As posições foram parametrizadas com auxílio do goniômetro e trena para manter a angulação de determinadas articulações e o alinhamento do corpo. O alinhamento do corpo proporcionou a capacidade de cada indivíduo reproduzir a posição orientada pelo avaliador, para que não ocorresse desvio do posicionamento. A coleta de dados com cada participante nos três decúbitos (dorsal, lateral e ventral) demandou em torno de 30 minutos. O registro da foto gerada pelo sensor do software foi realizado um minuto após cada posicionamento.

### Tamanho da Amostra

Neste estudo a unidade de análise foi o posicionamento em decúbito. Para o cálculo amostral foram considerados os parâmetros estimados no estudo piloto realizado previamente, com uma diferença na pressão entre as posições (dorsal, lateral, ventral) de 60% (tamanho do efeito de 0,60). Para um nível de significância de 5% e poder de 80%, estimou-se uma amostra de 45 indivíduos por grupo, com cada indivíduo sendo testado em 10 diferentes colchões o que resultou em um total de 450 avaliações. O cálculo foi realizado utilizando o programa GPower 3.1.9.7.

O tamanho da amostra, calculada a partir do resultado do piloto, foi superior à referência da fase I de estudo clínico, cujo produto desenvolvido é testado em um grupo de 20 a 100 voluntários sem comorbidades ou complicações, com o objetivo de avaliar a segurança, compatibilidade e eficácia do produto^(16)^.

### Métodos Estatísticos

Para a análise de dados foi utilizado o software SPSS versão 21.0. Foi realizada uma comparação da pressão total por decúbito, considerando o colchão C-CORE^®^, por meio de uma Análise de Variâncias (ANOVA) com um fator. Nesse caso, o desfecho considerado foi a pressão total e a variável de análise (grupo de estudo) o tipo de decúbito (dorsal, lateral e ventral). Essa análise foi estratificada por sexo e peso corporal. O teste de comparação múltipla de Tukey foi realizado quando detectadas diferenças significativas entre os grupos. Em todas as análises foi considerado um nível de significância de 5%.

### Aspectos Éticos

O estudo atendeu às Resoluções nº 466/12 do Conselho Nacional de Saúde. Foi aprovado pelo Comitê de Ética em Pesquisa sob o parecer nº 6.853.674, Certificado de Apresentação para Apreciação Ética (CAAE) 78828624.3.0000.5149. Ressalta-se que a participação foi condicionada à assinatura do Termo de Consentimento Livre Esclarecido pela participante.

Em respeito ao preconizado pela Ciência Aberta é informado que no momento os dados não estão disponíveis, uma vez que o estudo é fruto do Projeto de Pesquisa, Desenvolvimento e Inovação (PD&I), uma parceria público-privada com a empresa C-CORE Brasil.

## RESULTADOS

A Figura 1 apresenta o diagrama Consort de seleção dos participantes do estudo^(15)^. Foram recrutados 100 participantes para o estudo com alocação de 48. O estudo resultou em 464 avaliações em cada decúbito, considerando que esta é a unidade de análise, perfazendo o total de 1.392 decúbitos.

**Figura 1:** Diagrama *Consort*. Belo Horizonte, MG, Brasil, 2024.

Em relação as características dos participantes, 24 (50%) eram do sexo masculino e 24 (50%) feminino. O peso médio foi 69,7Kg (DP 24,3), com variação de 40,0Kg a 113,5Kg. A idade mínima foi 19 anos e máxima 53 anos, com média de 32,2 anos (DP 11,7).

Quando comparadas as médias de pressão exercida de acordo com os decúbitos dorsal, lateral e ventral (Figura 2), constatou- se pressão média na posição dorsal de 652,3 ± 30,2 (intervalo de confiança [IC] de 95%: 592,9–711,70), lateral de 1.522,2 ± 50,5(IC 95%:1.423,0–1.621,4) e ventral 313,2 ± 19,4 (IC 95%: 275,2–351,3).

**Figura 2:** Gráfico dos valores médios da pressão por posição.

Em relação as comparações múltiplas do total da pressão entre os decúbitos analisados, considerando os testes de comparações múltiplas (Tabela 1), houve diferença significativa entre eles (valor-p < 0,001).

**Tabela 1:** Comparações múltiplas da pressão entre os decúbitos analisados – Belo Horizonte, MG, Brasil, 2024.

Decúbito		Tamanho do efeito	Erro-padrão	Valor-p*	IC 95%^#^	
					Limite inferior	Limite superior
Dorsal	Lateral	–869,9	50,5	0,000	–988,4	–751,4
	Ventral	339,0	50,4	0,000	220,7	457,4
Lateral	Dorsal	869,9	50,5	0,000	751,4	988,4
	Ventral	1.208,9	50,5	0,000	1.090,5	1.327,5
Ventral	Dorsal	–339,0	50,4	0,000	–457,4	–220,7
	Lateral	–1.209,0	50,5	0,000	–1.327,5	–1.090,5

**Legenda:** *Teste Tukey.

^#^Intervalo de confiança 95% para a tamanho do efeito.

Quando estratificado por sexo (Figura 3), a diferença entre os decúbitos se mantém significativa (valores-p < 0,001). No sexo feminino, a média de pressão em decúbito dorsal foi de 540,08 ± 35,7 (IC 95%: 470,4–611,2). No decúbito lateral a pressão média foi 1.200,9 ± 61,7 (IC 95%: 1.079,2–1.322,6), enquanto no decúbito ventral foi 169,2 ± 15,5 (IC 95%: 138,7–199,6). Para o grupo do sexo masculino a pressão média no decúbito dorsal foi 761,3 ± 47,5 (IC 95%: 667,8–854,9). No decúbito lateral a média foi 1.842,0 ± 73,8 (IC 95%: 169,5–1.987,6) e no decúbito ventral foi 455,3 ± 32,7 (IC 95%: 390,9–519,7).

**Figura 3:** Valores médios da pressão por decúbito estratificado por sexo e peso.

A magnitude das diferenças entre a posição lateral e as demais posições mantem a diferença estatística em relação ao sexo. Também, quanto maior o peso, maiores as pressões no decúbito lateral em relação aos demais

Nos testes de comparações múltiplas (Tabela 2) houve diferença significativa entre todos os decúbitos para ambos os sexos. Observa-se, no entanto, que a magnitude das diferenças entre os decúbitos dorsal e lateral, e ventral e lateral são maiores entre os homens (efeito de 1.080,7 e 1.386,7, respectivamente) que entre as mulheres (efeito de 660,1 e 1.031,7, respectivamente).

**Tabela 2:** Comparações múltiplas da pressão (mmHg) entre os decúbitos por sexo e peso – Belo Horizonte, MG, Brasil, 2024.

Variável	Posição		Tamanho do efeito (mmHg)	Erro-padrão	Valor-p*	IC 95%^#^	
						Limite inferior (mmHg)	Limite superior (mmHg)
**Sexo**							
Feminino	Dorsal	Lateral	−660,1	59,6	0,000	−800,2	-520,0
		Ventral	371,6	59,6	0,000	231,5	511,7
	Lateral	Dorsal	660,1	59,6	0,000	520,0	800,2
		Ventral	1031,7	59,6	0,000	891,8	1.171,6
	Ventral	Dorsal	−371,6	59,6	0,000	−511,7	-231,5
		Lateral	−1031,7	59,6	0,000	−1171,6	-891,8
Masculino	Dorsal	Lateral	−1080,7	76,3	0,000	−1259,9	-901,5
		Ventral	306,0	76,1	0,000	127,2	484,8
	Lateral	Dorsal	1080,7	76,3	0,000	901,5	1.259,9
		Ventral	1386,7	76,4	0,000	1207,3	1.566,1
	Ventral	Dorsal	−306,0	76,1	0,000	−484,8	−127,2
		Lateral	−1386,7	76,4	0,000	−1.566,1	−1.207,3
**Peso**							
< 50 Kg	Dorsal	Lateral	-451,6	47,3	0,000	−563,0	−340,3
		Ventral	187,4	47,1	0,000	76,7	298,1
	Lateral	Dorsal	451,6	47,3	0,000	340,3	563,0
		Ventral	639,1	47,2	0,000	528,0	750,2
	Ventral	Dorsal	−187,4	47,1	0,000	−298,1	−76,7
		Lateral	−639,1	47,2	0,000	−750,2	−528,0
≥ 50Kg e ≤80Kg	Dorsal	Lateral	−879,9	62,4	0,000	−1.026,5	−733,3
		Ventral	370,4	62,5	0,000	223,5	517,3
	Lateral	Dorsal	879,9	62,4	0,000	733,3	1.026,5
		Ventral	1250,3	62,5	0,000	1.103,5	1.397,0
	Ventral	Dorsal	−370,4	62,5	0,000	−517,3	−223,5
		Lateral	−1250,3	62,5	0,000	−1.397,0	−1.103,5
> 80 Kg e ≤ 100 Kg	Dorsal	Lateral	−1244,1	165,9	0,000	−1.640,2	−847,9
		Ventral	323,1	164,4	0,128	−69,5	715,6
	Lateral	Dorsal	1244,1	165,9	0,000	847,9	1.640,2
		Ventral	1567,1	165,9	0,000	1.171,0	1.963,3
	Ventral	Dorsal	−323,1	164,4	0,128	−715,6	69,5
		Lateral	−1567,1	165,9	0,000	−1963,3	−1171,0
> 100 Kg e ≤ 180kg	Dorsal	Lateral	−1384,3	143,5	0,000	−1722,6	−1046,0
		Ventral	514,8	143,5	0,001	176,5	853,1
	Lateral	Dorsal	1384,3	143,5	0,000	1046,0	1722,6
		Ventral	1899,1	143,5	0,000	1560,7	2237,4
	Ventral	Dorsal	−514,8	143,5	0,001	−853,1	−176,5
		Lateral	−1899,1	143,5	0,000	−2237,4	−1560,7

**Legenda:** *Teste Tukey.

^#^Intervalo de confiança de 95% para média.

A diferença entre os decúbitos é também significativa (valores-p < 0,001) na estratificação por peso, com menores níveis de pressão para o decúbito ventral e maiores para o decúbito lateral, para as quatro categorias de peso (Tabela 2).

## DISCUSSÃO

Houve diferença significativa da pressão entre os decúbitos dorsal, lateral e ventral, com menores níveis de pressão para a posição ventral. O decúbito lateral foi a posição que gerou maiores pressões nas proeminências ósseas, principalmente na região trocantérica. Entender o resultado da pressão decorrente do posicionamento pode amparar estratégias para minimizar deformações nos tecidos, reduzindo os valores de pico de tensão e estresse ou diminuindo o tempo de exposição e, consequentemente, prevenindo a lesão por pressão.

Os resultados de uma revisão sistemática indicam que o reposicionamento mais frequente e o uso de uma equipe de rotação reduzem a incidência de lesão por pressão. No entanto, houve baixa certeza das evidências, por isso, os resultados devem ser interpretados com cautela^(17)^. Estes resultados aliados ao achado de que a posição lateral provoca maiores pressões nas proeminências ósseas de pessoas saudáveis, podem subsidiar os profissionais da prática clínica na definição do tempo de permanência do paciente neste decúbito. A tomada de decisão deve considerar a capacidade da superfície de suporte (colchão) em redistribuir a pressão.

Em condições normais, a pressão capilar máxima é de aproximadamente 20 mmHg e a pressão média tecidual varia entre 16 e 33 mmHg. No entanto, quando pressões superiores são desencadeadas por longos períodos do corpo na mesma posição, há um risco aumentado de evoluir para necrose tecidual. A compressão prolongada dos tecidos leva à redução do fluxo sanguíneo, ocasionando dano microcirculatório e diminuição na entrega de oxigênio, resultando em isquemia e lesão por pressão^(9)^. Quando comparadas as médias de pressão exercida de acordo com os decúbitos, constatou-se pressão média na posição ventral de 313,2 mmHg, dorsal de 652,3 mmHg e lateral de 1.522,2 mmHg. Estes valores são superiores à pressão capilar máxima. Por isso, é preciso garantir o regime de mudança de decúbito e superfície de suporte compatível com a necessidade.

Altas deformações do tecido resultam em danos celulares em um nível microscópico em apenas alguns minutos, embora possa levar horas de carga sustentada para que o dano se torne clinicamente visível. Danos superficiais à pele parecem ser causados principalmente por exposições excessivas de tensão. Contudo, lesões por pressão mais profundas resultam predominantemente de altas pressões em combinação com cisalhamento sobre proeminências ósseas^(18)^.

Nas últimas duas décadas tornou-se evidente que a deformação sustentada das células leva diretamente a danos e morte celular. As vias propostas incluem alterações no citoesqueleto e um aumento da permeabilidade das membranas celulares, excedendo o limiar para a homeostase celular normal. Sob alta deformação tecidual, quando o limiar de dano individual é excedido, acredita-se que esse processo seja rápido, em minutos^(19)^.

Considerando todas as camadas de tecido comprometidas, as evidências indicam que sinais de desenvolvimento de lesão por pressão podem ser detectáveis em todos os tecidos envolvidos, incluindo pele, gordura subcutânea ou músculo^(20)^ A localização anatômica, a morfologia (por exemplo, geometria óssea, espessura do tecido), as propriedades dos tecidos moles e rígidos, o grau de deformação do tecido, o microclima da pele, a tolerância individual e a capacidade de reparo determinam se os limiares de dano são excedidos ou não, levando a lesões por pressão mais profundas ou superficiais^(21)^.

Em 2005, pesquisadores investigaram quatro regimes preventivos envolvendo mudanças de decúbito frequentes (2, 3 horas) ou menos frequentes (4, 6 horas) em colchões diferentes. Durante 28 dias, foram utilizados quatro esquemas diferentes: mudança a cada 2 h em um colchão institucional padrão, mudança a cada 3 h em um colchão padrão, mudança a cada 4 h em um colchão de espuma viscoelástica e mudança a cada 6 h em um colchão de espuma viscoelástica. Estes grupos tinham 65 ou 67 pacientes. Os pesquisadores concluíram que fazer mudança de decúbito no colchão viscoelástico a cada 4 horas resultou em uma redução significativa no número de lesões por pressão^(22)^. Contudo, uma recente revisão sistemática da Cochrane evidenciou que o reposicionamento pode ser útil para reduzir o risco de lesões por pressão. Entretanto, sua eficácia depende de fatores, como a superfície de suporte utilizada e o estado clínico do paciente. Além disso, não foi possível determinar com certeza qual tempo é mais eficaz para o reposicionamento, a cada 2, 3 ou 4 horas. Inclusive, quando se trata do decúbito lateral, não há forte evidência apontando que a inclinação de 30° ou 90° seja uma estratégia efetiva para reduzir a pressão em áreas vulneráveis^(10)^.

No presente estudo, a magnitude das diferenças entre os decúbitos dorsal e lateral e, ventral e lateral são maiores entre os homens que entre as mulheres, isso pode ser justificado pela diferença anatômica da estrutura óssea de cada sexo. Esse fato tem relação com o resultado de um estudo que identificou que a inclinação ideal para reposicionamento pode depender do sexo e do índice de massa corporal^(8)^.

Em contrapartida, estudos mostram que o paciente posicionado no decúbito lateral, com um ângulo de 90° em relação a superfície de suporte por mais de 2 horas pode reduzir o fluxo sanguíneo a níveis próximos da anoxia, enquanto uma inclinação de 30° melhora a oxigenação e reduz complicações^(9)^. Embora o reposicionamento do paciente seja aplicado no cotidiano hospitalar, extra-hospitalar e recomendado por diretrizes de boas práticas, ainda há necessidade de estudos robustos sobre o melhor intervalo de tempo para o reposicionamento do paciente.

As diferenças anatômicas entre os homens e mulheres evidencia que homens podem estar mais predispostos a lesões por pressão devido à maior concentração de forças em áreas específicas da pelve. A pelve masculina possui um ângulo subpúbico mais fechado e acetábulos mais próximos, resultando em uma estrutura mais compacta e rígida. Essa configuração pode concentrar o peso corporal em áreas menores, como as tuberosidades isquiáticas, gerando maior pressão nessas regiões, especialmente durante a posição sentada ou decúbito lateral^(23)^. Embora o sexo possa influenciar nas características anatômicas e fisiológicas, deve-se considerar que ele não é um fator determinante isolado para o surgimento de lesões por pressão^(24)^. As divergências reforçam a necessidade de estudos sobre o tema.

Um estudo realizado com indivíduos saudáveis constatou que aqueles com obesidade exerceram maior pressão na região trocantérica a uma inclinação corporal de 30°, quando comparados a indivíduos com sobrepeso, peso normal e baixo peso, respectivamente^(8)^. O estudo realizado identificou que o peso corporal tem relação significativa com a pressão de interface no decúbito lateral. Quanto maior o peso, maior é a pressão nas proeminências ósseas. O achado apoia os enfermeiros na definição de tempo para o reposicionamento. Portanto, o tempo para manter o paciente na posição lateral deve ser menor para aqueles com maior peso corporal, uma vez que a possibilidade de ocorrência de lesão por pressão aumenta proporcionalmente com o aumento do peso. Diante desses achados, sugere-se a necessidade de considerar, além do fator sexo, a influência do peso corporal na pressão exercida, o que pode impactar na formulação de estratégias para a prevenção de lesões por pressão.

No estudo, a posição ventral foi aquela com menor pressão nas proeminências ósseas. O decúbito ventral, conhecido como posição prona, ficou em evidência no manejo da síndrome do desconforto respiratório agudo em pacientes com COVID-19 no período de pandemia. Ele trouxe como principal benefício a redução da mortalidade. Porém, essa posição também apresentou algumas desvantagens. Uma delas foi o aumento do risco da ocorrência da lesão por pressão em regiões corporais atípicas, devido ao contato contínuo com a superfície de suporte, como por exemplo face (testa, asa do nariz, bochecha, lábio, mento), peito, joelho, região pré-tibial e dedos do pé^(25)^.

Além do reposicionamento, a superfície de suporte deve ser ajustada para atender às necessidades individuais do paciente, considerando fatores como o estado da pele e a estratificação do risco de lesões. Porém, há falta de evidências que amparam que uma superfície de suporte seja indicada como padrão ouro na distribuição correta do peso e alívio da pressão^(26)^. Identificar a frequência ideal de mudança de decúbito e reposicionamento é fundamental para a prevenção de lesão por pressão em ambientes de saúde. Além disso, pesquisadores de enfermagem devem ser incentivados a realizar pesquisas adicionais para investigar os fatores que influenciam a eficácia da frequência de mudança no desenvolvimento de lesão por pressão^(12.)^.

Este é um estudo de desenho experimental fatorial sem randomização o que pode introduzir vieses, pois a alocação dos participantes nas diferentes posições não foi aleatória, o que pode afetar a validade interna. Como o estudo foi conduzido em um ambiente laboratorial com 10 colchões da mesma tecnologia, os achados podem não ser diretamente aplicáveis a outras populações, contextos clínicos ou superfícies de suporte diferentes. Destaca-se que a avaliação da pressão de interface foi realizada exclusivamente com o software do sensor SR Soft Vision®, o que pode limitar a comparação com outras tecnologias ou métodos de mensuração da interface paciente-colchão. O estudo analisou 1.392 posicionamentos obtidos de 48 participantes, o que pode comprometer a representatividade e a correlação entre medidas repetidas dentro do mesmo indivíduo. Embora o estudo tenha estratificado por idade, sexo e peso corporal, outras variáveis, como tônus muscular, índice de massa corporal ou distribuição da gordura corporal, não foram consideradas, o que pode influenciar os resultados.

A intervenção (tipo de decúbito) apresentou resultados relevantes para os profissionais, principalmente os de enfermagem, na definição do decúbito para o posicionamento dos pacientes em risco de lesão por pressão, especialmente para aqueles com comprometimento da mobilidade. Os achados amparam a prescrição de enfermagem quanto ao decúbito e recomendam que seja dada maior atenção ao decúbito lateral, por desencadear maior pressão na interface das regiões de proeminências ósseas.

Os resultados embasam a necessidade de revisar o tempo para o reposicionamento dos homens e aqueles com maior peso corporal, independentemente da superfície de suporte em uso. Por fim, indica-se que a equipe de enfermagem empregue escalas de avaliação de risco para lesão por pressão, estabeleça intervenções assertivas e monitore a ocorrência de lesão por pressão, como por exemplo, instrumentos que podem avaliar a qualidade e efetividade das abordagens na rotina de prevenção de lesão por pressão. Outra potencialidade do estudo é a utilização dos resultados para a revisão de protocolos de prevenção de lesão por pressão, com destaque para a revisão do tempo de reposicionamento considerando os decúbitos dorsal, lateral e ventral, conforme o escore de risco dos pacientes, sexo e peso corporal.

O estudo oferece subsídios para futuras investigações clínicas em cenário hospitalar, com o objetivo de avaliar, no contexto real, as pressões exercidas nos decúbitos lateral, ventral e dorsal de pacientes em risco para o desenvolvimento de lesões por pressão.

## CONCLUSÃO

O estudo refutou a hipótese que os decúbitos ventral, lateral e dorsal têm a mesma pressão nas proeminências ósseas (pressão de interface), uma vez que houve menores níveis de pressão para o decúbito ventral e maiores para o decúbito lateral em ambos os sexos e peso corporal. O fato sustenta a opção pelo decúbito ventral para os pacientes com restrição no reposicionamento. A magnitude das diferenças entre os decúbitos dorsal e lateral, ventral e lateral são maiores entre os homens e a magnitude das diferenças entre os decúbitos aumenta conforme a elevação do peso, isto é, quanto maior o peso, maior a pressão na posição lateral em relação às demais. Os achados reforçam a necessidade de rever protocolos de mudança de decúbito, minimizando os picos de pressão em áreas vulneráveis.

## Data Availability

Os dados que embasam este estudo são confidenciais e não estão disponíveis publicamente, pois derivam de um Projeto de Pesquisa, Desenvolvimento e Inovação (PD&I) realizado em parceria público-privada com a empresa C-CORE Brasil.

# Translation: Presión en las prominencias óseas según las posiciones decúbito dorsal, lateral y ventral: ensayo clínico

**DOI:** 10.1590/1980-220X-REEUSP-2025-0073es

**Authors:** Eline Lima Borges, Ana Luizi Perígolo Miranda, Perla Oliveira Soares de Souza, Mery Natali Silva Abreu, Josimare Aparecida Otoni Spira

**Affiliations:** 1 Universidade Federal de Minas Gerais, Escola de Enfermagem, Enfermagem Básica, Belo Horizonte, MG, Brasil.; 2 Universidade Federal de Minas Gerais, Escola de Enfermagem, Belo Horizonte, MG, Brasil.; 3 Universidade Federal de Minas Gerais, Escola de Enfermagem, Laboratório de Tecnologia e Inovação, Belo Horizonte, MG, Brasil.; 4 Universidade Federal de Minas Gerais, Escola de Enfermagem, Gestão em Saúde, Belo Horizonte, MG, Brasil.

**Keywords:** Úlcera por Presión, Presión, Posición Supina, Atención de Enfermería, Ensayo Clínico

## Abstract

**Objetivo:**

Identificar la presión en las prominencias óseas en las posiciones dorsal, lateral y ventral.

**Método:**

Estudio clínico de fase I, experimental y no aleatorio, que comparó las presiones de interfaz en las prominencias óseas en tres posiciones (dorsal, lateral y ventral) en individuos sanos. La presión se midió con el sensor SR Soft Vision® en todos los participantes, colocados en las tres posiciones y sobre 10 colchones C-CORE^®^. La comparación de la presión total por posición se realizó mediante ANOVA.

**Resultados:**

Se realizaron 448 evaluaciones en cada posición. La presión media fue de 652,3 (IC 95%: 592,9−711,7) en decúbito dorsal, 1522,2 (IC 95%: 1423,0−1621,4) en lateral y 313,2 (IC 95%: 275,2−351,3) en ventral. Al estratificar por sexo (p < 0,001), en las mujeres, la presión media fue de 540,08 en decúbito dorsal, 1200,9 en decúbito lateral y 169,2 en decúbito ventral. En los hombres, fue de 761,3 en decúbito dorsal, 1842,0 en decúbito lateral y 455,3 en decúbito ventral. La estratificación por peso también presentó diferencias significativas (p < 0,001) entre las decúbitos.

**Conclusión:**

Este estudio demostró una variación significativa en las presiones de interfaz entre las decúbitos dorsal, lateral y ventral. Registro en ReBEC: RBR-9vsvkn5.

## INTRODUCCIÓN

Las lesiones por presión son uno de los eventos adversos más frecuentes y costosos en los hospitales, pero son potencialmente evitables. Son reconocidas internacionalmente como un indicador importante de la calidad de la prestación de cuidados de salud^(1)^. Su prevención es uno de los objetivos de seguridad del paciente y responsabilidad del equipo multidisciplinar en todos los niveles de atención del sistema sanitario^(2)^. Cuando se desarrollan, el coste del tratamiento puede variar desde una cobertura protectora de las prominencias óseas hasta una superficie de soporte dinámica y puede alcanzar varios miles de euros, dólares o reales^(3)^.

Las lesiones por presión son un gran reto para la salud pública, con variaciones entre países en cuanto a su carga a nivel mundial. Los datos del *Global Burden of Disease Study* 2019 confirman que se ha producido una mejora de algunos indicadores entre 1990 y 2019. A nivel mundial, en 2019, el número de casos prevalentes de lesiones por presión fue de 0,85 millones, la prevalencia estandarizada por edad, la incidencia y las tasas de años vividos con discapacidad fueron de 11,3, 41,8 y 1,7 por cada 100 000 habitantes. La mayor carga de lesiones por presión se observó en pacientes de edad avanzada, especialmente en el grupo de 80 a 84 años para los hombres y de 85 a 89 para las mujeres^(4)^. Sin embargo, no se observaron diferencias estadísticamente significativas en la prevalencia, la incidencia y los años vividos con discapacidad entre hombres y mujeres. Se mantuvieron diferencias regionales significativas entre los años vividos con discapacidad, estandarizados por edad e índice sociodemográfico^(4)^.

Desde la perspectiva del paciente, las lesiones por presión causan pérdidas físicas, psicológicas, sociales y económicas. En términos de servicios de salud, contribuyen a pérdidas laborales y financieras significativas. Además, el costo de las estrategias de prevención de lesiones por presión mantiene diferencias estadísticamente significativas^(3)^. Un estudio de revisión sistemática sobre el impacto económico de las lesiones por presión en pacientes ingresados en unidades de terapia intensiva identificó que las cuatro principales fuentes de coste para la prevención eran: superficies de apoyo, cubiertas para prominencias óseas, recursos humanos, especialmente de enfermería, y equipos para la movilización^(5)^.

El reposicionamiento, en horarios individualizados, de todas las personas con riesgo de sufrir lesiones por presión se encuentra entre las medidas de prevención^(6)^. Esta acción tiene como objetivo reducir la duración y la magnitud de la presión ejercida sobre las zonas vulnerables del cuerpo y contribuir al confort, la higiene, la dignidad y la capacidad funcional de la persona^(6)^.

La exposición del tejido blando a deformaciones sostenidas y concentraciones de estrés, que normalmente se producen en las proximidades de las prominencias óseas, está relacionada con la posición del cuerpo (decúbito) sobre la superficie de apoyo. Las relaciones entre los niveles de concentración de estrés del tejido blando cerca de las prominencias óseas y el grado de implicación durante las posturas de soporte de peso son muy complejas y pueden estar relacionadas con la aparición de lesiones por presión. Estas interacciones dependen de las características anatómicas y geométricas del paciente, de la composición del material y de la estructura de la superficie de apoyo, además de la posición específica del cuerpo^(7)^. Algunos estudios han evaluado la intensidad de la presión en las prominencias óseas bajo la influencia de la inclinación del cuerpo^(8)^ y su reposicionamiento^(9,10)^.

No hay pruebas claras sobre cuál es la mejor posición y el mejor programa de reposicionamiento. Una revisión constató que, en general, la calidad de los estudios es limitada^(11)^. Un metaanálisis no identificó pruebas concluyentes sobre la eficacia de diferentes frecuencias y posiciones de cambio de decúbito en la prevención de lesiones por presión. Sin embargo, los cambios cada 3 o 4 horas pueden ser más económicos que cada 2 horas. En cuanto a las posiciones de inclinación (30° frente a 90°), los resultados también fueron inconclusos^(10)^. Aunque el reposicionamiento es necesario para prevenir las lesiones por presión, cambiar la posición con menos frecuencia puede ser una realidad en la práctica clínica, dadas las propiedades de las nuevas tecnologías de colchones y la falta de concordancia en la evidencia sobre el reposicionamiento^(11)^.

La posición habitual de los pacientes para distribuir la presión es la posición dorsal plana, que ayuda a minimizar el cizallamiento y a prevenir las lesiones por presión en las prominencias sacro/coccígeas^(12)^. Sin embargo, los pacientes no deben permanecer todo el tiempo en esta posición. También se les coloca en posición lateral y, en casos de insuficiencia respiratoria grave, en posición prona.

Se considera que la mayoría de los centros hospitalarios no disponen de equipos sofisticados para la prevención, como colchones de rotación asistida^(13)^ o sensores portátiles para orientar el reposicionamiento de los pacientes^(14)^. Una estrategia popular consiste en la rotación y el reposicionamiento frecuentes de los pacientes^(12)^. Por lo tanto, el conocimiento sobre el valor de la presión en las prominencias óseas según la posición decúbito puede contribuir a la adopción de estrategias de rotación y movimiento de los pacientes para prevenir o reducir las lesiones por presión, especialmente en aquellos con inmovilidad o dificultades de movimiento.

Ante la insuficiencia de pruebas sobre la intensidad de la presión en las prominencias óseas y su relación con la posición decúbito, surgió la siguiente pregunta de investigación: ¿cuál es la presión en las prominencias óseas según la posición decúbito (dorsal, lateral y ventral)? La respuesta a la pregunta del estudio puede respaldar la revisión de los protocolos de prevención de lesiones por presión en el manejo del reposicionamiento de pacientes en riesgo. En vista de lo anterior, el estudio tiene como objetivo identificar la presión en las prominencias óseas en las decúbitos dorsal, lateral y ventral.

## MÉTODO

### Diseño del Estudio

Se trata de la fase I de un estudio clínico, de tipo experimental factorial, no aleatorio, comparativo, sin enmascaramiento, para dilucidar la hipótesis: las decúbitos ventral, lateral y dorsal tienen la misma presión en las prominencias óseas (presión de interfaz). Para la comunicación del estudio se han tenido en cuenta los supuestos del documento *Consolidated Standards of Reporting Trials* (CONSORT)^(15)^. El estudio está registrado en el Registro Brasileño de Ensayos Clínicos (ReBEC), con el identificador primario RBR-9vsvkn5.

### Participantes

Los criterios de elegibilidad fueron: participantes de ambos sexos, mayores de 18 años, presencia de todos los miembros del cuerpo, independencia de movimiento, que permitiera a la persona moverse de forma autónoma y sin restricciones, y peso de hasta 180 kg. Los criterios de exclusión fueron: informe de incontinencia urinaria o fecal, presencia de desviación acentuada de la columna vertebral a la inspección, enfermedades dermatológicas de tipo descamativo, heridas cutáneas o cicatrices en la región glútea o trocantérica.

El estudio se llevó a cabo en el Laboratorio de Tecnología e Innovación (LABTEC C-CORE/UFMG), ubicado en la Escuela de Enfermería de la Universidad Federal de Minas Gerais. En el LABTEC se creó un escenario similar al de una unidad de hospitalización con una cama hospitalaria de la marca Qualitas Plus™ Paramount bed - serie A5, con colchón C-CORE^®^, cuya materia prima es producida por la empresa C-CORE Brasil. El colchón está compuesto por una manta tridimensional reticulada de filamentos de polietileno de alta densidad y cadena larga (3D LOOP), flexible, cuya característica aireada puede potenciar el control del microclima de la piel (temperatura y humedad). La organización de los filamentos crea una estructura que permite un alto nivel de ventilación. El ambiente durante la recolección de datos se mantuvo a una temperatura de 23 °C.

El reclutamiento se llevó a cabo entre enero y junio de 2024, mediante una invitación en la que los interesados debían rellenar un formulario en *Google Forms* con información personal y responder a preguntas según los criterios de elegibilidad. Además, se programaba el día y la hora para la recolección de datos.

### Resultado

El estudio requirió tres intervenciones: decúbito dorsal, decúbito ventral y decúbito lateral, formando tres grupos distintos para la medición del resultado, la presión de interfaz en las prominencias óseas.

La recolección de datos se realizó entre junio y diciembre de 2024. Los participantes recibieron explicaciones sobre el estudio y tuvieron la oportunidad de aclarar cualquier duda. Además, se midió el peso corporal.

La variable dependiente (resultado) fue la presión de interfaz y la variable independiente el tipo de decúbito (dorsal, lateral y ventral). También se tuvieron en cuenta las siguientes variables: edad, sexo y peso (en kilos) categorizado en < 50 kg, ≥ 50 kg y ≤ 80 kg, > 80 kg y ≤ 100 kg, > 100 kg y ≤ 180 kg.

Para evaluar la presión de interfaz se utilizó un sensor SR Soft Vision^®^, que es una manta compuesta por minúsculas células que captan la magnitud de la presión y el área de contacto con la superficie del cuerpo de la persona, convirtiéndola en datos mediante un software compatible con Windows^®^. La manta se colocó sobre cada colchón y el cable del sensor, conectado al ordenador, captaba la imagen. Para este estudio se consideraron los puntos de presión iguales o superiores a 79 mmHg, clasificados como elevados. Para calcular la presión total se sumaron todos los puntos de presión elevada.

Para el estudio se utilizaron 10 tipos de colchones C-CORE^®^ de 15 cm de altura, con diferentes densidades. El resultado de la prueba piloto en decúbito dorsal, lateral y ventral, con los 10 colchones, permitió considerar los colchones como variable única, sin categorías.

Cada participante vistió ropa ligera, tipo *leggings* con camiseta, sin bolsillos, lazos ni costuras para evitar puntos de presión. Se colocó a la persona en posición supina sin almohada y se evaluó la presión, especialmente en las zonas de prominencias óseas. Todos los participantes fueron colocados en las tres posiciones decúbito y sobre los 10 colchones de la tecnología C-CORE^®^, respetando la secuencia de programación en LABTEC.

En la posición dorsal se midió la presión en las regiones de la cabeza, el dorso, el cubital posterior, el coxis y el talón. En posición lateral, se midieron las regiones de la cara, la espalda, la trocantérica, la rótula y el lateral del pie. En posición ventral (prona), se evaluaron las regiones de la oreja, la cara, la parte posterior del codo, el pecho, la pelvis, la rótula y los dedos del pie. Las posiciones se parametrizaron con la ayuda de un goniómetro y una cinta métrica para mantener la angulación de determinadas articulaciones y la alineación del cuerpo. La alineación del cuerpo permitió que cada individuo reprodujera la posición indicada por el evaluador, para que no se produjeran desviaciones en el posicionamiento. La recolección de datos con cada participante en las tres posiciones decúbito (dorsal, lateral y ventral) requirió alrededor de 30 minutos. El registro de la foto generada por el sensor del software se realizó un minuto después de cada posicionamiento.

### Tamaño de la Muestra

En este estudio, la unidad de análisis fue la posición en decúbito. Para el cálculo de la muestra se consideraron los parámetros estimados en el estudio piloto realizado previamente, con una diferencia en la presión entre las posiciones (dorsal, lateral, ventral) del 60% (tamaño del efecto de 0,60). Para un nivel de significación del 5% y una potencia del 80%, se estimó una muestra de 45 individuos por grupo, y cada individuo fue evaluado en 10 colchones diferentes, lo que dio un total de 450 evaluaciones. El cálculo se realizó utilizando el programa GPower 3.1.9.7.

El tamaño de la muestra, calculado a partir del resultado del estudio piloto, fue superior a la referencia de la fase I del estudio clínico, en el que el producto desarrollado se prueba en un grupo de 20 a 100 voluntarios sin comorbilidades ni complicaciones, con el objetivo de evaluar la seguridad, la compatibilidad y la eficacia del producto^(16)^.

### Métodos Estadísticos

Para el análisis de los datos se utilizó el software SPSS versión 21.0. Se realizó una comparación de la presión total por decúbito, considerando el colchón C-CORE^®^, mediante un análisis de varianza (ANOVA) con un factor. En este caso, el resultado considerado fue la presión total y la variable de análisis (grupo de estudio) el tipo de decúbito (dorsal, lateral y ventral). Este análisis se estratificó por sexo y peso corporal. Se realizó la prueba de comparación múltiple de Tukey cuando se detectaron diferencias significativas entre los grupos. En todos los análisis se consideró un nivel de significación del 5%.

### Aspectos Éticos

El estudio cumplió con las Resoluciones n.º 466/12 del Consejo Nacional de Salud. Fue aprobado por el Comité de Ética en Investigación bajo el dictamen n.º 6.853.674, Certificado de Presentación para Apreciación Ética (CAAE) 78828624.3.0000.5149. Cabe destacar que la participación estuvo condicionada a la firma del Término de Consentimiento Libre y Esclarecido por parte de la participante.

En cumplimiento de lo preconizado por la Ciencia Abierta, se informa que, en este momento, los datos no están disponibles, ya que el estudio es fruto del Proyecto de Investigación, Desarrollo e Innovación (PD&I), una asociación público- privada con la empresa C-CORE Brasil.

## RESULTADOS

La Figura 1 presenta el diagrama Consort de selección de los participantes en el estudio^(15)^. Se reclutaron 100 participantes para el estudio, con una asignación de 48. El estudio dio como resultado 464 evaluaciones en cada decúbito, considerando que esta es la unidad de análisis, lo que suma un total de 1392 decúbitos.

**Figura 1:** Diagrama *Consort*. Belo Horizonte, MG, Brasil, 2024.

En cuanto a las características de los participantes, 24 (50%) eran hombres y 24 (50%) mujeres. El peso medio fue de 69,7 kg (DE 24,3), con una variación de 40,0 kg a 113,5 kg. La edad mínima fue de 19 años y la máxima de 53 años, con una media de 32,2 años (DE 11,7).

Al comparar los promedios de presión ejercida según las posiciones dorsal, lateral y ventral (Figura 2), se observó una presión media en posición dorsal de 652,3 ± 30,2 (intervalo de confianza [IC] del 95%: 592,9–711,70), lateral de 1522,2 ± 50,5 (IC del 95%: 1423,0–1621,4) y ventral de 313,2 ± 19,4 (IC del 95%: 275,2–351,3).

**Figura 2:** Gráfico de los valores medios de la presión por posición.

En relación con las comparaciones múltiples de la presión total entre las decúbitos analizadas, teniendo en cuenta las pruebas de comparaciones múltiples (Tabla 1), se observó una diferencia significativa entre ellas (valor p < 0,001).

**Tabla 1:** Comparaciones múltiples de la presión entre los decúbitos analizados. Belo Horizonte, MG, Brasil, 2024.

Decúbito		Tamaño del efecto	Error estándar	Valor-p*	IC 95%^#^	
					Límite inferior	Límite superior
Dorsal	Lateral	–869,9	50,5	0,000	–988,4	–751,4
	Ventral	339,0	50,4	0,000	220,7	457,4
Lateral	Dorsal	869,9	50,5	0,000	751,4	988,4
	Ventral	1.208,9	50,5	0,000	1.090,5	1.327,5
Ventral	Dorsal	–339,0	50,4	0,000	–457,4	–220,7
	Lateral	–1.209,0	50,5	0,000	–1.327,5	–1.090,5

**Clave:** *Test Tukey.

^#^Intervalo de confianza 95% para el tamaño del efecto.

Cuando se estratificó por sexo (Figura 3), la diferencia entre las posiciones decúbito se mantuvo significativa (valores p < 0,001). En las mujeres, la presión media en decúbito dorsal fue de 540,08 ± 35,7 (IC 95%: 470,4–611,2). En decúbito lateral, la presión media fue de 1200,9 ± 61,7 (IC 95%: 1079,2–1322,6), mientras que en decúbito ventral fue de 169,2 ± 15,5 (IC 95%: 138,7–199,6). Para el grupo masculino, la presión media en decúbito dorsal fue de 761,3 ± 47,5 (IC 95%: 667,8–854,9). En decúbito lateral, la media fue de 1.842,0 ± 73,8 (IC 95%: 169,5–1.987,6) y en decúbito ventral fue de 455,3 ± 32,7 (IC 95%: 390,9–519,7).

**Figura 3:** Valores medios de la presión por decúbito estratificados por sexo y peso.

La magnitud de las diferencias entre la posición lateral y las demás posiciones mantiene la diferencia estadística en relación con el sexo. Además, cuanto mayor es el peso, mayores son las presiones en decúbito lateral en relación con las demás.

En las pruebas de comparaciones múltiples (Tabla 2) se observó una diferencia significativa entre todos los decúbitos para ambos sexos. Sin embargo, se observa que la magnitud de las diferencias entre las decúbitos dorsal y lateral, y ventral y lateral es mayor entre los hombres (efecto de 1080,7 y 1386,7, respectivamente) que entre las mujeres (efecto de 660,1 y 1031,7, respectivamente). La diferencia entre las posiciones decúbito también es significativa (valores p < 0,001) en la estratificación por peso, con niveles de presión más bajos para la posición decúbito ventral y más altos para la posición decúbito lateral, para las cuatro categorías de peso (Tabla 2).

**Tabla 2:** Comparaciones múltiples de la presión (mmHg) entre las posiciones decúbito por sexo y peso. Belo Horizonte, MG, Brasil, 2024.

Variable	Posición		Tamaño del efecto (mmHg)	Error estándar	Valor-p*	IC 95%^#^	
						Límite inferior (mmHg)	Límite superior (mmHg)
**Sexo**							
Femenino	Dorsal	Lateral	−660,1	59,6	0,000	−800,2	−520,0
		Ventral	371,6	59,6	0,000	231,5	511,7
	Lateral	Dorsal	660,1	59,6	0,000	520,0	800,2
		Ventral	1031,7	59,6	0,000	891,8	1.171,6
	Ventral	Dorsal	−371,6	59,6	0,000	−511,7	−231,5
		Lateral	−1031,7	59,6	0,000	−1171,6	−891,8
Masculino	Dorsal	Lateral	−1080,7	76,3	0,000	−1259,9	−901,5
		Ventral	306,0	76,1	0,000	127,2	484,8
	Lateral	Dorsal	1080,7	76,3	0,000	901,5	1.259,9
		Ventral	1386,7	76,4	0,000	1207,3	1.566,1
	Ventral	Dorsal	−306,0	76,1	0,000	−484,8	−127,2
		Lateral	−1386,7	76,4	0,000	−1.566,1	−1.207,3
**Peso**							
< 50 Kg	Dorsal	Lateral	−451,6	47,3	0,000	−563,0	−340,3
		Ventral	187,4	47,1	0,000	76,7	298,1
	Lateral	Dorsal	451,6	47,3	0,000	340,3	563,0
		Ventral	639,1	47,2	0,000	528,0	750,2
	Ventral	Dorsal	−187,4	47,1	0,000	−298,1	−76,7
		Lateral	−639,1	47,2	0,000	−750,2	−528,0
≥ 50Kg e ≤80Kg	Dorsal	Lateral	−879,9	62,4	0,000	−1.026,5	−733,3
		Ventral	370,4	62,5	0,000	223,5	517,3
	Lateral	Dorsal	879,9	62,4	0,000	733,3	1.026,5
		Ventral	1250,3	62,5	0,000	1.103,5	1.397,0
	Ventral	Dorsal	−370,4	62,5	0,000	−517,3	−223,5
		Lateral	−1250,3	62,5	0,000	−1.397,0	−1.103,5
> 80 Kg e ≤ 100 Kg	Dorsal	Lateral	−1244,1	165,9	0,000	−1.640,2	−847,9
		Ventral	323,1	164,4	0,128	−69,5	715,6
	Lateral	Dorsal	1244,1	165,9	0,000	847,9	1.640,2
		Ventral	1567,1	165,9	0,000	1.171,0	1.963,3
	Ventral	Dorsal	−323,1	164,4	0,128	−715,6	69,5
		Lateral	−1567,1	165,9	0,000	−1963,3	−1171,0
> 100 Kg e ≤ 180kg	Dorsal	Lateral	−1384,3	143,5	0,000	−1722,6	−1046,0
		Ventral	514,8	143,5	0,001	176,5	853,1
	Lateral	Dorsal	1384,3	143,5	0,000	1046,0	1722,6
		Ventral	1899,1	143,5	0,000	1560,7	2237,4
	Ventral	Dorsal	−514,8	143,5	0,001	−853,1	−176,5
		Lateral	−1899,1	143,5	0,000	−2237,4	−1560,7

**Clave:** *Test Tukey.

^#^Intervalo de confianza de 95% para media.

## DISCUSIÓN

Se observó una diferencia significativa en la presión entre las posiciones decúbito dorsal, lateral y ventral, con niveles de presión más bajos en la posición ventral. La posición decúbito lateral fue la que generó mayores presiones en las prominencias óseas, principalmente en la región trocantérica. Comprender el resultado de la presión derivada del posicionamiento puede respaldar estrategias para minimizar las deformaciones en los tejidos, reduciendo los valores máximos de tensión y estrés o disminuyendo el tiempo de exposición y, en consecuencia, previniendo las lesiones por presión.

Los resultados de una revisión sistemática indican que el reposicionamiento más frecuente y el uso de un equipo de rotación reducen la incidencia de lesiones por presión. Sin embargo, la certeza de las pruebas fue baja, por lo que los resultados deben interpretarse con cautela^(17)^. Estos resultados, junto con el hallazgo de que la posición lateral provoca mayores presiones en las prominencias óseas de las personas sanas, pueden ayudar a los profesionales de la práctica clínica a definir el tiempo de permanencia del paciente en esta posición. La toma de decisiones debe tener en cuenta la capacidad de la superficie de apoyo (colchón) para redistribuir la presión.

En condiciones normales, la presión capilar máxima es de aproximadamente 20 mmHg y la presión tisular media varía entre 16 y 33 mmHg. Sin embargo, cuando se producen presiones superiores durante largos periodos de tiempo con el cuerpo en la misma posición, existe un mayor riesgo de que se desarrolle necrosis tisular. La compresión prolongada de los tejidos provoca una reducción del flujo sanguíneo, lo que da lugar a daños microcirculatorios y a una disminución del suministro de oxígeno, lo que provoca isquemia y lesiones por presión^(9)^. Al comparar los promedios de presión ejercida según las decúbitos, se observó una presión media en posición ventral de 313,2 mmHg, dorsal de 652,3 mmHg y lateral de 1522,2 mmHg. Estos valores son superiores a la presión capilar máxima. Por lo tanto, es necesario garantizar el régimen de cambio de decúbito y la superficie de apoyo compatible con la necesidad.

Las deformaciones elevadas del tejido provocan daños celulares a nivel microscópico en solo unos minutos, aunque pueden pasar horas de carga sostenida antes de que el daño sea clínicamente visible. Los daños superficiales de la piel parecen estar causados principalmente por una exposición excesiva a la tensión. Sin embargo, las lesiones por presión más profundas se deben principalmente a presiones elevadas en combinación con cizallamiento sobre prominencias óseas^(18)^.

En las últimas dos décadas se ha hecho evidente que la deformación sostenida de las células conduce directamente al daño y la muerte celular. Las vías propuestas incluyen alteraciones en el citoesqueleto y un aumento de la permeabilidad de las membranas celulares, superando el umbral de la homeostasis celular normal. Bajo una alta deformación tisular, cuando se supera el umbral de daño individual, se cree que este proceso es rápido, en cuestión de minutos^(19)^.

Teniendo en cuenta todas las capas de tejido afectadas, las pruebas indican que los signos de desarrollo de una lesión por presión pueden ser detectables en todos los tejidos implicados, incluyendo la piel, la grasa subcutánea o el músculo^(20)^. La localización anatómica, la morfología (por ejemplo, la geometría ósea, el grosor del tejido), las propiedades de los tejidos blandos y rígidos, el grado de deformación del tejido, el microclima de la piel, la tolerancia individual y la capacidad de reparación determinan si se superan o no los umbrales de daño, lo que da lugar a lesiones por presión más profundas o superficiales^(21)^.^.^

En 2005, los investigadores estudiaron cuatro regímenes preventivos que implicaban cambios de decúbito frecuentes (2, 3 horas) o menos frecuentes (4, 6 horas) en diferentes colchones. Durante 28 días, se utilizaron cuatro esquemas diferentes: cambio cada 2 h en un colchón institucional estándar, cambio cada 3 h en un colchón estándar, cambio cada 4 h en un colchón de espuma viscoelástica y cambio cada 6 h en un colchón de espuma viscoelástica. Estos grupos tenían 65 o 67 pacientes. Los investigadores concluyeron que cambiar la posición del paciente en el colchón viscoelástico cada 4 horas dio como resultado una reducción significativa en el número de lesiones por presión^(22)^. Sin embargo, una reciente revisión sistemática de Cochrane ha puesto de manifiesto que el reposicionamiento puede ser útil para reducir el riesgo de lesiones por presión. No obstante, su eficacia depende de factores como la superficie de apoyo utilizada y el estado clínico del paciente. Además, no se ha podido determinar con certeza cuál es el intervalo más eficaz para el reposicionamiento, si cada 2, 3 o 4 horas. Incluso en el caso de la decúbito lateral, no hay pruebas sólidas que indiquen que la inclinación de 30° o 90° sea una estrategia eficaz para reducir la presión en las zonas vulnerables^(10)^.

En el presente estudio, la magnitud de las diferencias entre las decúbitos dorsal y lateral, y ventral y lateral es mayor entre los hombres que entre las mujeres, lo que puede justificarse por la diferencia anatómica de la estructura ósea de cada sexo. Este hecho está relacionado con el resultado de un estudio que identificó que la inclinación ideal para el reposicionamiento puede depender del sexo y del índice de masa corporal^(8)^.

Por otro lado, los estudios muestran que el paciente colocado en decúbito lateral, con un ángulo de 90° con respecto a la superficie de apoyo durante más de 2 horas, puede reducir el flujo sanguíneo a niveles cercanos a la anoxia, mientras que una inclinación de 30° mejora la oxigenación y reduce las complicaciones^(9)^. Aunque el reposicionamiento del paciente se aplica en el ámbito hospitalario y extrahospitalario y se recomienda en las directrices de buenas prácticas, aún se necesitan estudios sólidos sobre el mejor intervalo de tiempo para el reposicionamiento del paciente.

Las diferencias anatómicas entre hombres y mujeres evidencian que los hombres pueden ser más propensos a las lesiones por presión debido a la mayor concentración de fuerzas en áreas específicas de la pelvis. La pelvis masculina tiene un ángulo subpúbico más cerrado y acetábulos más cercanos, lo que da como resultado una estructura más compacta y rígida. Esta configuración puede concentrar el peso corporal en áreas más pequeñas, como las tuberosidades isquiáticas, generando una mayor presión en estas regiones, especialmente durante la posición sentada o decúbito lateral^(23)^. Aunque el sexo puede influir en las características anatómicas y fisiológicas, hay que tener en cuenta que no es un factor determinante por sí solo para la aparición de lesiones por presión^(24)^. Las divergencias refuerzan la necesidad de realizar más estudios sobre el tema.

Un estudio realizado con individuos sanos constató que aquellos con obesidad ejercían mayor presión en la región trocantérica con una inclinación corporal de 30°, en comparación con individuos con sobrepeso, peso normal y bajo peso, respectivamente^(8)^. El estudio realizado identificó que el peso corporal tiene una relación significativa con la presión de interfase en decúbito lateral. Cuanto mayor es el peso, mayor es la presión en las prominencias óseas. El hallazgo respalda a los enfermeros en la definición del tiempo para el reposicionamiento. Por lo tanto, el tiempo para mantener al paciente en posición lateral debe ser menor para aquellos con mayor peso corporal, ya que la posibilidad de que se produzcan lesiones por presión aumenta proporcionalmente con el aumento del peso. Ante estos hallazgos, se sugiere la necesidad de considerar, además del factor sexo, la influencia del peso corporal en la presión ejercida, lo que puede influir en la formulación de estrategias para la prevención de lesiones por presión.

En el estudio, la posición ventral fue la que presentó menor presión en las prominencias óseas. La decúbito ventral, conocida como posición prona, se destacó en el manejo del síndrome de dificultad respiratoria aguda en pacientes con COVID-19 durante la pandemia. Su principal beneficio fue la reducción de la mortalidad. Sin embargo, esta posición también presentó algunas desventajas. Una de ellas fue el aumento del riesgo de lesiones por presión en regiones corporales atípicas, debido al contacto continuo con la superficie de apoyo, como por ejemplo la cara (frente, alas de la nariz, mejillas, labios, mentón), el pecho, las rodillas, la región pretibial y los dedos de los pies^(25)^.

Además del reposicionamiento, la superficie de apoyo debe ajustarse para satisfacer las necesidades individuales del paciente, teniendo en cuenta factores como el estado de la piel y la estratificación del riesgo de lesiones. Sin embargo, no hay pruebas que respalden que una superficie de apoyo sea la norma de referencia para la distribución correcta del peso y el alivio de la presión^(26)^. Identificar la frecuencia ideal de cambio de decúbito y reposicionamiento es fundamental para la prevención de lesiones por presión en entornos de salud. Además, se debe alentar a los investigadores de enfermería a realizar investigaciones adicionales para investigar los factores que influyen en la eficacia de la frecuencia de cambio en el desarrollo de lesiones por presión^(12.)^.

Este es un estudio de diseño experimental factorial sin aleatorización, lo que puede introducir sesgos, ya que la asignación de los participantes a las diferentes posiciones no fue aleatoria, lo que puede afectar a la validez interna. Dado que el estudio se llevó a cabo en un entorno de laboratorio con 10 colchones de la misma tecnología, los resultados pueden no ser directamente aplicables a otras poblaciones, contextos clínicos o superficies de apoyo diferentes. Cabe destacar que la evaluación de la presión de interfaz se realizó exclusivamente con el software del sensor SR Soft Vision®, lo que puede limitar la comparación con otras tecnologías o métodos de medición de la interfaz paciente-colchón. El estudio analizó 1392 posicionamientos obtenidos de 48 participantes, lo que puede comprometer la representatividad y la correlación entre medidas repetidas dentro del mismo individuo. Aunque el estudio se estratificó por edad, sexo y peso corporal, no se tuvieron en cuenta otras variables, como el tono muscular, el índice de masa corporal o la distribución de la grasa corporal, lo que puede influir en los resultados.

La intervención (tipo de decúbito) presentó resultados relevantes para los profesionales, principalmente los de enfermería, en la definición del decúbito para el posicionamiento de pacientes en riesgo de lesiones por presión, especialmente para aquellos con movilidad comprometida. Los hallazgos respaldan la prescripción de enfermería en cuanto a la decúbito y recomiendan que se preste mayor atención a la decúbito lateral, ya que provoca una mayor presión en la interfaz de las regiones de prominencias óseas.

Los resultados respaldan la necesidad de revisar el tiempo para el reposicionamiento de los hombres y de aquellos con mayor peso corporal, independientemente de la superficie de apoyo utilizada. Por último, se recomienda que el equipo de enfermería utilice escalas de evaluación del riesgo de lesiones por presión, establezca intervenciones asertivas y supervise la aparición de lesiones por presión, por ejemplo, instrumentos que puedan evaluar la calidad y la eficacia de los enfoques en la rutina de prevención de lesiones por presión. Otra posibilidad del estudio es la utilización de los resultados para revisar los protocolos de prevención de lesiones por presión, con especial atención a la revisión del tiempo de reposicionamiento teniendo en cuenta las posiciones dorsal, lateral y ventral, según la puntuación de riesgo de los pacientes, el sexo y el peso corporal.

El estudio ofrece información para futuras investigaciones clínicas en el ámbito hospitalario, con el objetivo de evaluar, en un contexto real, las presiones ejercidas en las decúbitos lateral, ventral y dorsal de pacientes con riesgo de desarrollar lesiones por presión.

## CONCLUSIÓN

El estudio refutó la hipótesis de que las decúbitos ventral, lateral y dorsal ejercen la misma presión sobre las prominencias óseas (presión de interfaz), ya que se observaron niveles de presión más bajos para la decúbito ventral y más altos para la decúbito lateral en ambos sexos y pesos corporales. Este hecho respalda la opción de la decúbito ventral para los pacientes con restricción en el reposicionamiento. La magnitud de las diferencias entre las decúbitos dorsal y lateral, ventral y lateral es mayor entre los hombres, y la magnitud de las diferencias entre las decúbitos aumenta con el aumento del peso, es decir, cuanto mayor es el peso, mayor es la presión en la posición lateral en relación con las demás. Los hallazgos refuerzan la necesidad de revisar los protocolos de cambio de decúbito, minimizando los picos de presión en las zonas vulnerables.

## Data Availability

Los datos que respaldan este estudio son confidenciales y no están disponibles públicamente, ya que se derivan de un Proyecto de Investigación, Desarrollo e Innovación (I+D+i) realizado mediante una asociación público-privada con la empresa C-CORE Brasil.
